# Evaluating generalizability of oncology trial results to real-world patients using machine learning-based trial emulations

**DOI:** 10.1038/s41591-024-03352-5

**Published:** 2025-01-03

**Authors:** Xavier Orcutt, Kan Chen, Ronac Mamtani, Qi Long, Ravi B. Parikh

**Affiliations:** 1 Navajo Indian Health Service, Chinle, AZ USA; 2 Department of Biostatistics, Harvard University, Boston, MA USA; 3 Perelman School of Medicine, University of Pennsylvania, Philadelphia, PA USA; 4 Penn Center for Cancer Care Innovation, Abramson Cancer Center, Philadelphia, PA USA; 5 Department of Biostatistics, Epidemiology and Informatics, University of Pennsylvania, Philadelphia, PA USA; 6 Emory University School of Medicine, Atlanta, GA USA; 7 Winship Cancer Institute, Atlanta, GA USA

**Keywords:** Randomized controlled trials, Prognostic markers, Prognosis

## Abstract

Randomized controlled trials (RCTs) evaluating anti-cancer agents often lack generalizability to real-world oncology patients. Although restrictive eligibility criteria contribute to this issue, the role of selection bias related to prognostic risk remains unclear. In this study, we developed TrialTranslator, a framework designed to systematically evaluate the generalizability of RCTs for oncology therapies. Using a nationwide database of electronic health records from Flatiron Health, this framework emulates RCTs across three prognostic phenotypes identified through machine learning models. We applied this approach to 11 landmark RCTs that investigated anti-cancer regimens considered standard of care for the four most prevalent advanced solid malignancies. Our analyses reveal that patients in low-risk and medium-risk phenotypes exhibit survival times and treatment-associated survival benefits similar to those observed in RCTs. In contrast, high-risk phenotypes show significantly lower survival times and treatment-associated survival benefits compared to RCTs. Our results were corroborated by a comprehensive robustness assessment, including examinations of specific patient subgroups, holdout validation and semi-synthetic data simulation. These findings suggest that the prognostic heterogeneity among real-world oncology patients plays a substantial role in the limited generalizability of RCT results. Machine learning frameworks may facilitate individual patient-level decision support and estimation of real-world treatment benefits to guide trial design.

## Abstract (web summary)

Machine-learning-based and population-level electronic health record data–based emulation of landmark phase 3 randomized controlled trials (RCTs) in oncology across a range of cancer and risk types showed that the median overall survival treatment benefit for real-world patients would be, on average, 3 months lower than in RCTs, with high-risk phenotypes faring worse.

## Main

The limited generalizability of results from randomized controlled trials (RCTs) evaluating anti-cancer agents has been a longstanding concern for patients, clinicians and regulators. Real-world survival associated with anti-cancer therapies is often significantly lower than that reported in RCTs^1–5^, with some studies showing a median reduction of 6 months in median overall survival (mOS)^6^. For novel agents such as checkpoint inhibitors, observational studies suggest that real-world patients experience both decreased overall survival and reduced survival benefits relative to the standard of care^7^.

Restrictive eligibility criteria in RCTs are frequently cited as a cause of this lack of generalizability, resulting in a study population that poorly represents the broader oncology patient community^8^. Approximately one in five real-world oncology patients are ineligible for a phase 3 trial^9^. However, restrictive eligibility criteria alone are unlikely to fully explain the generalizability gap. A study simulating various eligibility criteria combinations in advanced non-small cell lung cancer (aNSCLC) trials found little variation in survival hazard ratios (HRs) for treatment^10^. This suggests that other factors may be at play. An alternative explanation is that physicians selectively recruit patients with better prognoses, specifically those who are younger with fewer comorbidities, irrespective of eligibility criteria. Additionally, preferential recruitment based on factors, such as race or socioeconomic status (SES), both of which are linked to prognosis, may also contribute to this issue^11^. Consequently, real-world patients likely have more heterogeneous prognoses than RCT participants^12^.

Considering the varied survival outcomes among real-world oncology patients, accurately translating phase 3 trial results is crucial for treatment decision-making and advance care planning. Although RCTs often include subgroup analyses to evaluate treatment effect heterogeneity, these are typically underpowered, focus on single patient characteristics and rarely adjust for multiple testing or pre-specify hypotheses^13,14^. Improved methods for translating phase 3 trials to real-world patients with multiple varying characteristics are necessary.

The combination of well-curated electronic health record (EHR) data and machine learning (ML) phenotyping could help identify real-world patient groups whose treatment effects align with published RCT results. The recent availability of population-level EHR data enriched with clinical factors and molecular biomarkers offers the potential for improved trial emulation through enhanced baseline feature balancing between treatment arms^15^. ML models leveraging these granular datasets may uncover subtle patterns and relationships that may not be apparent through conventional analysis, potentially revealing more nuanced prognostic groups^16,17^.

Although robust frameworks for trial emulation exist^18^, ML-based adjustment for patient prognosis has not been widely implemented. Previous studies used ML in trial emulation to simulate various combinations of eligibility criteria on treatment effect^10^, create digital twins of RCTs using EHR data^19^ and predict individualized treatment effects in patients with heart failure^20^. However, a comprehensive approach to systematically evaluate RCT generalizability across different prognostic groups in oncology is lacking. Here we introduce TrialTranslator, a framework that systematically emulates phase 3 oncology trials across ML-identified prognostic phenotypes to uncover treatment effect heterogeneity in real-world patients.

## Results

### Overview of TrialTranslator

**Fig. 1:**
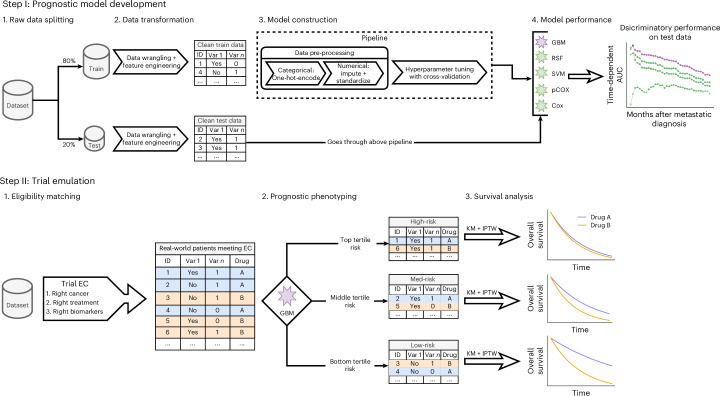
TrialTranslator workflow. The TrialTranslator workflow consists of two steps. Step I involves the development of prognostic models tailored to specific cancer types, designed to predict the risk of patient-level mortality from time of metastatic diagnosis. The GBM emerged as the top-performing model for all four cancers and was selected for the trial emulation phase. Step II encompasses the trial emulation process, which unfolds in three parts: (1) eligibility matching: real-world patients are selected who meet key eligibility criteria (EC) from landmark clinical trials; (2) prognostic phenotyping: within each trial, selected patients are stratified based on prognostic risk scores using the cancer-specific GBM; and (3) survival analysis: IPTW-adjusted Kaplan–Meier (KM) survival curves are calculated for each prognostic phenotype to evaluate treatment effect and compare to respective RCT results.

TrialTranslator is a framework that uses ML models to risk stratify real-world oncology patients into distinct prognostic phenotypes before emulating landmark phase 3 trials to assess the result generalizability (Fig. 1). We applied it to 11 landmark phase 3 RCTs that demonstrated a survival benefit favoring treatment over control. These RCTs investigated anti-cancer regimens considered standards of care and recommended in clinical guidelines, at the time of analysis, for the four most prevalent advanced solid malignancies in the USA: aNSCLC, metastatic breast cancer (mBC), metastatic prostate cancer (mPC) and metastatic colorectal cancer (mCRC).

The framework consists of two sequential steps. In step I, prognostic model development, multiple cancer-specific prognostic models are constructed to predict patient mortality risk from the time of metastatic diagnosis. The top-performing model is then selected for use in step II.

Step II, trial emulation, consists of three distinct parts. First, eligibility matching involves identifying real-world patients who received either the treatment or control regimens and meet the key eligibility criteria from the landmark RCTs. Next, prognostic phenotyping stratifies these patients within each trial into low-risk, medium-risk and high-risk phenotypes using mortality risk scores calculated from the top-performing ML model from step I. Finally, survival analysis assesses the treatment effect for each phenotype by calculating restricted mean survival time (RMST) and mOS, derived from inverse probability of treatment weighted (IPTW)-adjusted Kaplan–Meier survival curves. The results are then compared to those reported in the respective RCTs, highlighting potential differences in treatment effects across patient risk groups in real-world settings.

### Data source and trial selection

This study used the nationwide Flatiron Health EHR-derived database (https://flatiron.com/real-world-evidence), which is sourced from approximately 280 cancer clinics across the USA, for both prognostic model development and trial emulation. The database is a longitudinal repository containing de-identified patient-level structured and unstructured data. The distribution of cancers, along with the demographics and clinical characteristics of patients in this database, is similar to those in the National Cancer Institute’s Surveillance, Epidemiology, and End Results (SEER) population-based cancer registry^21^.

Our study focused specifically on aNSCLC, mBC, mPC and mCRC owing to their significant representation in the USA and in the Flatiron Health database. The final cohort comprised 68,483 patients with aNSCLC, 31,677 patients with mBC, 18,927 patients with mPC and 34,315 patients with mCRC (Table 1). These patients were diagnosed with advanced or metastatic disease between 2011 and 2022, with about 70% diagnosed before 2019.

**Table 1:** Baseline characteristics of patients with advanced cancer in the Flatiron Health dataset

Characteristic		aNSCLC	mBC	mPC	mCRC
*n*		68,483	31,677	18,927	34,315
Age at metastatic diagnosis, median (IQR)		70 (62–76)	64 (54–73)	74 (66–78)	65 (55–74)
Male sex, *n* (%)		35,811 (52.3)	375 (1.2)	18,927 (100.0)	18,876 (55.0)
Race, *n* (%)	White	46,653 (68.1)	20,395 (64.4)	11,619 (61.4)	21,706 (63.3)
	Black	5,832 (8.5)	3,742 (11.8)	1,923 (10.2)	3,774 (11.0)
	Asian	1,756 (2.6)	672 (2.1)	256 (1.4)	968 (2.8)
	Other	6,564 (9.6)	3,671 (11.6)	2,991 (15.8)	4,219 (12.3)
Region, *n* (%)	Northeast	11,896 (17.4)	4,446 (14.0)	n/a	4,861 (14.2)
	South	27,281 (39.8)	11,289 (35.6)	n/a	12,975 (37.8)
	Midwest	9,843 (14.4)	3,802 (12.0)	n/a	4,128 (12.0)
	West	10,171 (14.9)	4,882 (15.4)	n/a	5,498 (16.0)
Practice type, *n* (%)	Community	61,870 (90.3)	26,002 (82.1)	16,134 (85.2)	29,246 (85.2)
	Academic	6,613 (9.7)	5,271 (16.6)	2,470 (13.1)	4,752 (13.8)
Insurance, *n* (%)	Commercial	27,701 (40.4)	13,143 (41.5)	6,929 (36.6)	13,765 (40.1)
	Medicare	23,515 (34.3)	11,088 (35.0)	10,254 (54.2)	12,340 (36.0)
	Medicaid	3,281 (4.8)	2,746 (8.7)	705 (3.7)	2,794 (8.1)
ECOG score	0	10,482 (15.3)	6,813 (21.5)	2,680 (14.2)	7,261 (21.2)
	1	14,357 (21.0)	4,908 (15.5)	2,343 (12.4)	6,200 (18.1)
	≥2	7,358 (10.7)	2,363 (7.5)	1,049 (5.5)	2,323 (6.8)
	Unknown	36,286 (53.0)	17,593 (55.5)	12,855 (67.9)	18,531 (54.0)
Year of metastatic diagnosis	2011–2014	24,112 (35.2)	9,880 (31.2)	3,553 (18.8)	6,960 (20.3)
	2015–2018	30,871 (45.1)	12,259 (38.7)	8,236 (43.5)	15,470 (45.1)
	2019–2022	13,500 (19.7)	9,538 (30.1)	7,138 (37.7)	11,885 (34.6)
Weight loss > 10%, *n* (%)		6,305 (9.2)	1,856 (5.9)	429 (2.3)	3,140 (9.2)
Labs, *n* (%)	Creatinine > 2 mg dl^⁻1^	794 (1.2)	268 (0.8)	422 (2.2)	461 (1.3)
	Hemoglobin < 9 g dl^⁻1^	1,541 (2.3)	702 (2.2)	507 (2.7)	1,803 (5.3)
	Total bilirubin > 3 mg dl^⁻1^	86 (0.1)	188 (0.6)	17 (0.1)	353 (1.0)
	Albumin < 3 g dl^⁻1^	2,778 (4.1)	615 (1.9)	212 (1.1)	1,399 (4.1)
	Missing above labs	21,251 (31.0)	11,037 (34.8)	10,291 (54.4)	12,033 (35.1)
Censored, *n* (%)		19,401 (28.3)	13,305 (42.0)	9,115 (48.2)	12,971 (37.8)

Patient characteristics at time of diagnosis of aNSCLC, mBC, mPC or mCRC. Features were selected at the time nearest to advanced or metastatic diagnosis. ‘Missing above labs’ indicates absence of all four key laboratory values at time of advanced diagnosis: creatinine, hemoglobin, total bilirubin and albumin. IQR, interquartile range; n/a, not applicable.

**Extended Data Fig. 1:**
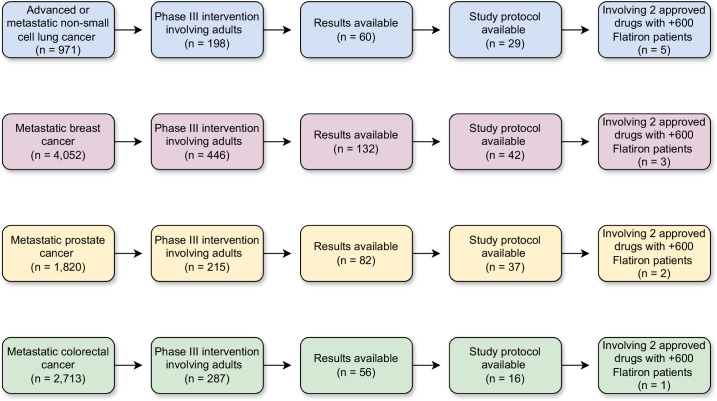
Selection process for RCT emulation. Selection of the 11 trials for emulation in advanced non-small cell lung cancer, metastatic breast cancer, metastatic prostate cancer, and metastatic colorectal cancer queried from ClinicalTrials.gov. Drugs were considered approved if they were included in the National Comprehensive Cancer Network guidelines.

Phase 3 RCTs pertaining to the four cancers were considered for emulation if they demonstrated an overall or progression-free survival benefit for the treatment arm, if they involved treatment regimens that were standard of care as of January 2023 and if the Flatiron Health database contained at least 600 patients meeting the key eligibility criteria for the respective RCT to ensure sufficient statistical power. This selection process resulted in the inclusion of 11 RCTs: five for aNSCLC, three for mBC, two for mPC and one for mCRC (Extended Data Fig. 1). These RCTs encompass a range of treatment modalities, including genotype-directed therapies, immune checkpoint inhibitors and hormone-based treatments, primarily focusing on first-line therapies for metastatic disease.

### Prognostic model development

Our primary modeling objective was to predict mortality from the time of metastatic diagnosis. To achieve this, we developed supervised survival-based ML models tailored to each cancer type. These models were optimized for predictive performance at specific timepoints: 1 year from metastatic diagnosis for aNSCLC and 2 years from metastatic diagnosisfor mBC, mCRC and mPC. These timepoints were selected to align with the mOS for each cancer type in the Flatiron Health database.

The ML models included a gradient boosting survival model (GBM)^22^, a random survival forest^23^, a survival linear support vector machine^24^ and three variations of a penalized Cox (pCox) proportional hazards model^25^. Additionally, we constructed a Cox proportional hazards model, inspired by retrospective studies for each cancer type^26–29^, as a benchmark for comparison with the ML models.

**Fig. 2:**
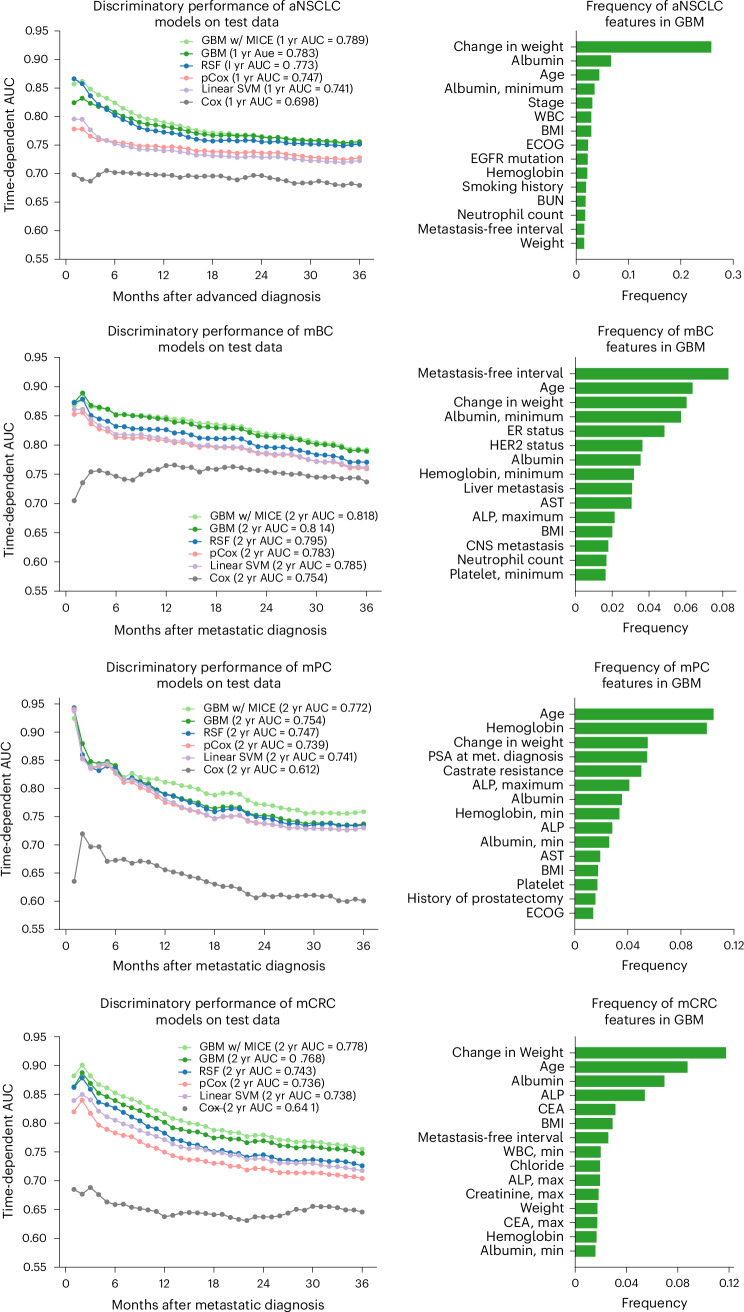
Discriminatory performance of models on test data. The figure illustrates the discriminatory performance of various ML models on a test set at different timepoints after diagnosis of aNSCLC, mBC, mPC and mCRC. The gradient boosting survival model with multiple imputation chained equations (GBM w/ MICE) performed best across cancer types at 1 or 2 years after advanced diagnosis, closely followed by the gradient boosting survival model with median imputation (GBM). ML models outperformed the Cox model, with ensemble models (random survival forest (RSF) and GBM) surpassing linear-based models (pCox and linear support vector machine (SVM)). ALP, alkaline phosphatase; AST, aspartate aminotransferase; BMI, body mass index; BUN, blood urea nitrogen; CEA, carcinoembryonic antigen; CNS, central nervous system; ER, estrogen receptor; PSA, prostate-specific antigen; WBC, white blood cell; yr, year.

Across all four cancer types, the GBM consistently demonstrated superior discriminatory performance on the test set in terms of the time-dependent area under the receiver operating characteristic curve (AUC) for 1-year and 2-year overall survival after advanced or metastatic diagnosis (Fig. 2 and Supplementary Table 1). For example, in aNSCLC, the 1-year survival AUC was 0.783 for the GBM compared to 0.689 for the Cox model based on the Lung Cancer Prognostic Index, a validated prognostic index derived from prospective data^26^. For mBC, mPC and mCRC, the 2-year AUCs were 0.814, 0.754 and 0.768, respectively, with the GBM outperforming the corresponding Cox models. Key features contributing to the predictive value of the GBM included age, weight loss, Eastern Cooperative Oncology Group (ECOG) performance status, cancer biomarkers and serum markers of frailty, such as albumin and hemoglobin.

### Trial emulation stratified by phenotype

In the primary analysis, eligibility for emulated trials was contingent upon patients meeting three key eligibility criteria: (1) having the correct cancer type, (2) receiving the treatment of interest at the appropriate line of therapy, and (3) possessing the relevant biomarker status at the time of treatment. Cohort sizes for the emulated trials ranged from 1,200 to 30,000 patients, with an average of 4,000 patients per trial (Supplementary Tables 2–12).

Patients meeting trial eligibility criteria then underwent prognostic phenotyping using the cancer-specific GBM from step I. The model calculated mortality risk scores for each patient, representing their likelihood of death by time *t*, with higher scores indicating a greater risk. These scores were used to rank individuals into the high-risk (top tertile), medium-risk (middle tertile) and low-risk (bottom tertile) phenotypes. Patients in the high-risk phenotype tended to be older, have an ECOG performance status greater than 1 and exhibit adverse laboratory values, such as higher creatinine, higher total bilirubin and lower hemoglobin levels, compared to both RCT populations and lower-risk phenotypes (Supplementary Tables 2–12).

**Extended Data Fig. 2:**
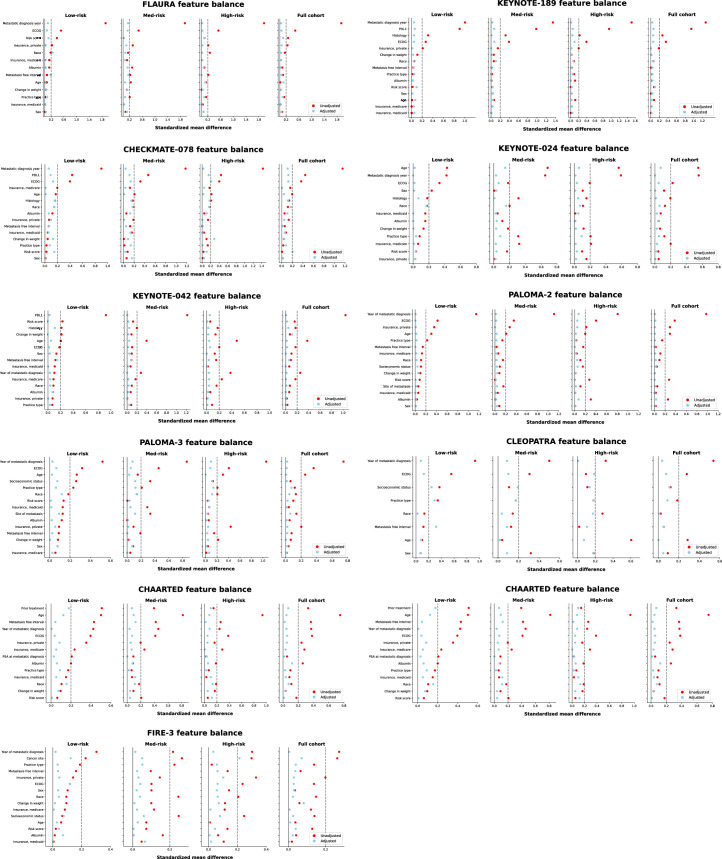
Feature balance for treatment and control groups across prognostic phenotypes. Absolute standardized mean differences for patient features in the propensity score model. Differences are shown before (unadjusted) and after (adjusted) applying inverse probability of treatment weighting. The vertical dashed line at 0.2 indicates the threshold for acceptable balance between treatment groups.

IPTW was applied to each phenotype to balance relevant features between treatment and control arms. The IPTW calculation accounted for demographic information, area-level SES, insurance status, cancer characteristics, ECOG performance status, cancer-specific biomarkers, albumin, weight change at time of metastatic diagnosis and mortality risk score. After adjustment, the absolute standardized mean difference between treatment and control arms was typically less than 20% (Extended Data Fig. 2). Time zero for the emulated trials was set as the start of the line of therapy.

To assess the quality of trial emulation, we estimated the treatment effect for the entire cohort meeting strict RCT eligibility criteria by calculating an HR from a Cox-IPTW model. Using a previously established framework^18^, trial emulation quality was assessed based on three criteria: (1) statistical significance, defined as the HR for the emulated trial favoring treatment and the 95% confidence interval (CI) being on the same side of the null value; (2) 95% CI agreement, defined as the HR for the emulated trial falling within the 95% CI of the RCT result; and (3) standardized difference agreement, defined as a standardized difference of less than 1.96 between the HR of the emulated trial and the RCT.

Eleven trials were emulated across the cancer types in the Flatiron Health database using strict eligibility criteria. In nine of the eleven emulated trials, the HR significantly favored the treatment arm (Extended Data Table 1). Five of the eleven emulated trials had HRs within the 95% CI of the RCT estimates, with a standardized difference within 1.96. Compared with the RCT results, emulated trial HRs were attenuated by an average of 35%. The mOS in treatment and control arms of emulated trials averaged 13% lower and 3% higher, respectively, than RCT reports (Extended Data Table 2). The difference in mOS between arms was, on average, 3.0 months shorter in the emulated trials than in the RCTs.

**Extended Data Fig. 3:**
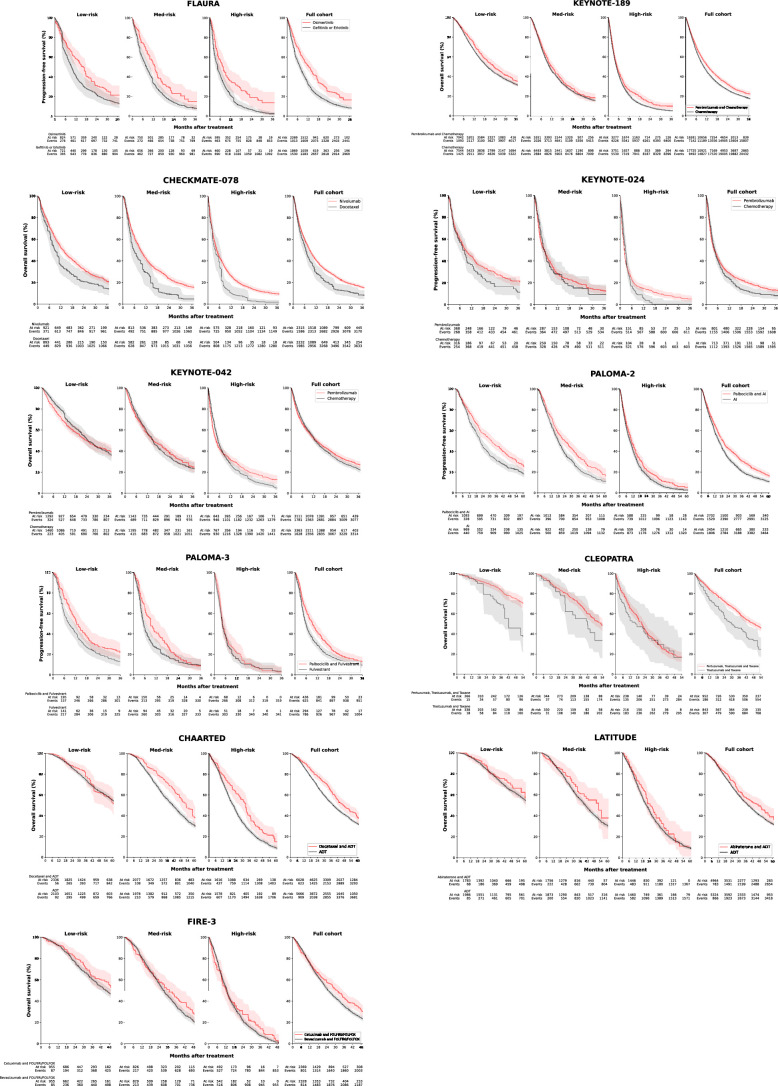
IPTW Kaplan-Meier survival curves. Inverse probability of treatment-weighted (IPTW) Kaplan-Meier survival curves for emulated trials. Solid lines show estimated survival probabilities. Shaded areas represent 95% confidence intervals, estimated via bootstrapping.

After establishing trial emulation quality, patients meeting key eligibility criteria were stratified into phenotypes, and the treatment effect for each phenotype was estimated using RMSTs and mOS. RMST was calculated approximately 1 year beyond the RCT-reported mOS to allow adequate separation of survival curves. RMST was chosen over HR owing to potential violations of the proportional hazards assumption, particularly in checkpoint inhibitor trials and high-risk phenotypes^15,30^. mOS was used as an additional treatment effect metric for direct comparison with RCT results. Both RMST and mOS were estimated non-parametrically using IPTW-adjusted Kaplan–Meier survival curves (Extended Data Fig. 3). For RCTs with progression-free survival as the primary outcome, restricted mean progression-free survival time and median progression-free survival (mPFS) were calculated instead.

After prognostic phenotyping of patients meeting key eligibility criteria, the difference in RMST was statistically significant in six of the eleven emulated trials for low-risk phenotypes, compared with five of eleven for medium-risk or high-risk phenotypes (Extended Data Table 3). Among emulated trials with significant RMST differences, low-risk and medium-risk phenotypes more frequently exhibited a difference greater than 3 months, a standard threshold of clinically meaningful benefit in oncology RCTs^31^, compared with high-risk phenotypes. A similar pattern was observed with mOS: only three of the eleven emulated trials in low-risk and medium-risk phenotypes failed to achieve a greater-than-3-month mOS, compared with seven of the eleven in high-risk phenotypes (Table 2).

**Table 2:** mOS times across prognostic phenotypes

Cancer	Trial	mOS in months (95% CI)											
		Low-risk			Medium-risk			High-risk			RCT		
		Treatment	Control	Δ Arms	Treatment	Control	Δ Arms	Treatment	Control	Δ Arms	Treatment	Control	Δ Arms
aNSCLC	FLAURA^a^	16.8 (13.1–18.0)	9.8 (6.6–12.4)	7.0	13.2 (10.2–14.7)	8.3 (6.7–10.2)	4.9	7.8 (4.9–10.5)	4.5 (3.4–5.8)	3.3	18.9 (15.2–21.4)	10.2 (9.6–11.1)	8.7
	KEYNOTE-189	23.9 (20.9–27.4)	19.8 (19.3–20.7)	4.0	12.4 (10.6–14.1)	11.0 (10.6–11.4)	1.4	5.1 (4.6–5.8)	4.7 (4.5–5.0)	0.4	22.0 (19.5–24.5)	10.6 (8.7–13.6)	11.4
	CHECKMATE-078	13.5 (11.9–14.4)	9.0 (6.7–11.3)	4.5	9.4 (8.6–10.4)	5.8 (4.3–9.4)	3.5	4.9 (4.4–5.5)	4.1 (2.5–6.4)	0.8	12.0 (10.4–14.0)	9.6 (7.6–11.2)	2.4
	KEYNOTE-024^a^	9.2 (7.7–11.1)	8.4 (5.5–9.8)	0.8	5.3 (4.0–6.2)	5.3 (4.2–7.7)	0.1	2.2 (2.0–2.4)	2.8 (2.1–3.4)	−0.6	10.3 (6.7–n/a)	6.0 (4.2–6.2)	4.3
	KEYNOTE-042	24.2 (20.4–29.4)	24.8 (21.5–29.0)	−0.6	15.4 (12.9–19.9)	15.0 (13.0–17.7)	0.4	4.3 (3.7–5.0)	5.4 (4.7–6.3)	−1.1	16.7 (13.9–19.7)	12.1 (11.3–13.3)	4.6
mBC	PALOMA-2^a^	32.0 (27.4–35.9)	21.9 (20.2–24.3)	10.2	24.0 (20.3–28.6)	18.3 (16.6–20.2)	5.7	10.8 (9.8–12.6)	9.3 (8.4–10.7)	1.6	27.6 (22.4–30.3)	14.5 (12.3–17.1)	13.1
	PALOMA-3^a^	13.8 (11.2–17.2)	8.6 (6.4–11.8)	5.1	9.4 (6.5–13.7)	5.4 (4.7–6.6)	4.0	4.6 (3.8–6.1)	4.3 (3.7–5.2)	0.3	9.5 (9.2–11.0)	4.6 (3.5–5.6)	4.9
	CLEOPATRA	90.6 (80.8–n/a)	51.9 (34.1–76.3)	38.7	53.1 (47.4–63.9)	42.2 (23.7–60.1)	10.9	21.6 (18.7–24.4)	17.5 (5.5–43.7)	4.1	57.1 (50.0–72.0)	40.8 (36.0–48.0)	16.3
mPC	CHAARTED	65.5 (52.1–n/a)	67.8 (62.9–72.5)	−2.2	53.0 (47.3–60.6)	40.7 (38.9–43.3)	12.3	33.4 (27.7–35.0)	21.7 (20.5–23.0)	11.7	57.6	44.0	13.6
	LATITUDE	n/a	68.2 (63.5–73.8)	n/a	52.6 (36.7–n/a)	40.7 (38.7–42.6)	11.9	25.4 (21.0–33.3)	21.4 (20.3–22.9)	4.0	53.3 (48.2–n/a)	36.5 (33.5–40.0)	16.8
mCRC	FIRE-3	53.0 (44.6–79.3)	45.7 (41.8–49.7)	7.3	31.4 (25.8–38.7)	27.4 (25.6–28.9)	3.9	12.9 (9.2–16.1)	12.8 (12.0–13.6)	0.1	33.1 (24.5–39.4)	25.6 (22.7–28.6)	7.5

mOS in months for treatment and control arms for a variety of emulated trials across prognostic phenotypes in patients with aNSCLC, mBC, mPC or mCRC; Δ Arms denotes the difference in survival time between treatment and control arms. Note that the CHAARTED results for the RCT did not publish CIs, and the LATITUDE low-risk treatment arm did not reach mOS.

^a^Trials reporting mPFS instead of mOS.

**Fig. 3:**
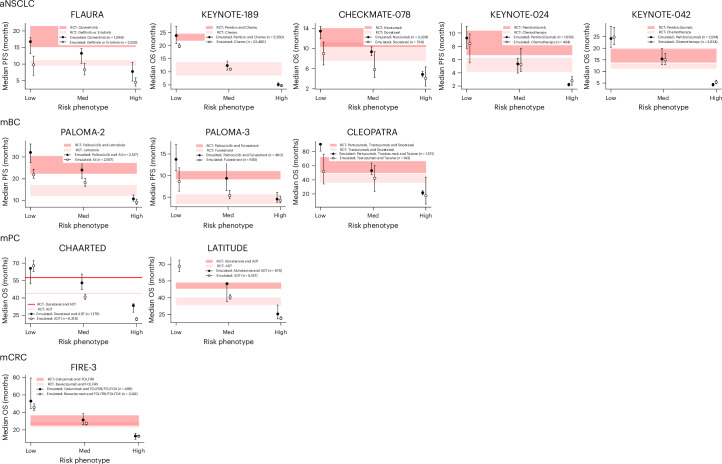
Median survival times for prognostic phenotypes versus RCT. These plots compare mOS and mPFS estimates with 95% CIs between emulated trials and original RCTs for patients with aNSCLC, mBC, mPC or mCRC. Emulated trial outcomes are stratified by prognostic phenotype. RCT results are displayed as red horizontal bars indicating the 95% CI. Median survival times are derived from IPTW Kaplan–Meier survival curves (Extended Data Fig. 3). Sample sizes for treatment and control arms of the full cohort in the emulated trial are included in the key of each plot. Note that the CHAARTED RCT did not report mOS 95% CIs; upper 95% CIs for treatment arms were not reached in the KEYNOTE-024 and LATITUDE RCTs; and the mOS for the treatment arm was not reached in the low-risk phenotype of the emulated LATITUDE trial. ADT, androgen deprivation therapy; AI, aromatase inhibitor.

**Fig. 4:**
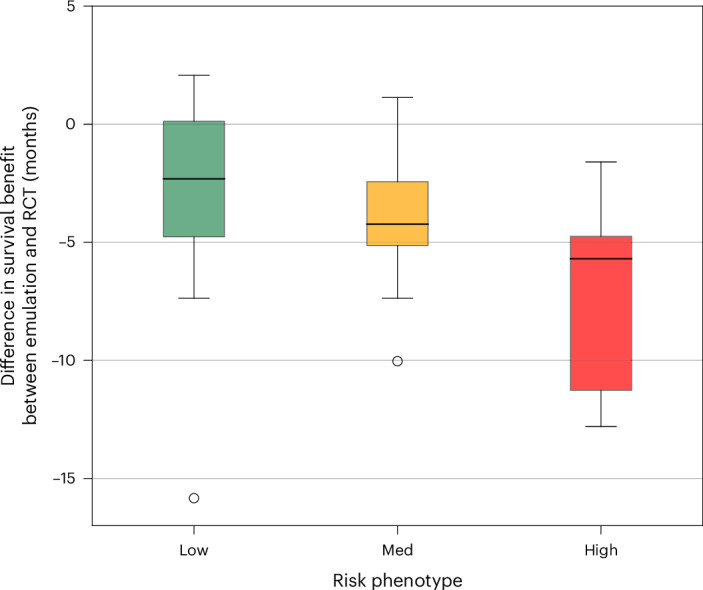
Comparison of survival benefit between trial emulation and RCT. Box plots compare treatment-associated survival benefit between 11 emulated trials and their corresponding RCT, stratified by prognostic phenotype. Survival benefit is defined as the difference in mOS between the treatment and control arms in months. Box plot elements: central line (median survival benefit); box hinges (25th and 75th percentiles); whiskers (extend to the smallest and largest values within 1.5 times the interquartile range from the hinge); and individual points (data beyond whiskers). Note that one individual point in the low-risk group (CLEOPATRA, 22.4 months) is not shown in the plot to better visualize the distribution of the remaining data. Zero indicates survival benefit matching RCT estimate. Compared with RCT predictions, low-risk phenotypes showed a 2.3-month shorter survival benefit; medium-risk phenotypes showed a 4.2-month shorter survival benefit; and high-risk phenotypes showed a 5.7-month shorter survival benefit. LATITUDE was not included in the calculation for the low-risk phenotype because mOS was not reached. For FLAURA, KEYNOTE-024, PALOMA-2 and PALOMA-3, survival benefit is based on mPFS rather than mOS.

**Extended Data Fig. 4:**
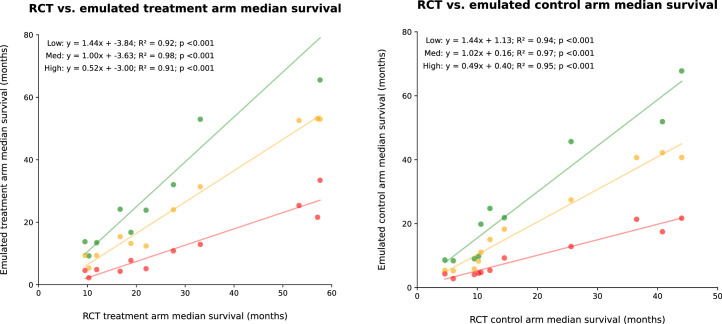
Survival outcome comparison between RCTs and emulated trials across prognostic phenotypes. Scatter plots comparing survival results between RCTs and emulated trials for treatment and control arms. Survival is median overall or progression-free survival, depending on the RCT primary outcome. Green, orange, and red dots represent low-, medium-, and high-risk phenotypes, respectively. Lines of best fit for low- and medium-risk phenotypes have slopes ≥1, indicating similar or better survival than RCT estimates; high-risk phenotypes have slopes <1, suggesting lower survival. R² values and corresponding p-values (F-statistic) provided for each phenotype, indicating strength and significance of linear relationships.

When comparing emulated trial outcomes with the RCT results, low-risk and medium-risk phenotypes showed survival times more aligned with RCT results than high-risk phenotypes. In about half of the emulated trials, the point estimate for treatment mOS in low-risk and medium-risk phenotypes fell within the RCT 95% CI (Fig. 3). However, for high-risk phenotypes, the treatment mOS point estimate averaged 62% lower than the RCT result and fell below the RCT 95% CI in all emulated trials (Fig. 3 and Extended Data Fig. 4). The mOS difference between treatment and control arms increasingly deviated from RCT results as phenotype risk increased. The median mOS difference was 2.3 months less (95% CI: −5.4 to 0.2) for low-risk phenotypes, 4.2 months less (95% CI: −5.4 to −1.3) for medium-risk phenotypes and 5.7 months less (95% CI: −11.5 to −4.6) for high-risk phenotypes compared to RCT results (Fig. 4).

Special attention was given to emulated trials involving checkpoint inhibitor agents (for example, KEYNOTE-189, CHECKMATE-078, KEYNOTE-024 and KEYNOTE-042) owing to their tendency for delayed treatment effects compared with traditional cancer therapies^32^. To account for this, survival probability at timepoints beyond mOS was calculated^33^. Both low-risk and high-risk phenotypes achieved a statistically significant difference in RMST in two or four emulated trials, with a similar magnitude of difference of approximately 1.5 months (Extended Data Table 3). Despite this apparent similarity in treatment effect, survival time remained substantially greater and closer to RCT results in low-risk phenotypes. For these phenotypes, the probability of survival at 1 year or 2 years in the checkpoint inhibitor arm was, on average, 6% higher than RCT results, whereas, for high-risk phenotypes, it was, on average, 60% lower (Supplementary Table 13).

### Robustness assessments

The robustness of the TrialTranslator results was assessed through three types of analyses: sensitivity, validation and a semi-synthetic data simulation. For the sensitivity analyses, we examined the impact of different imputation strategies on GBM discriminatory performance and performed two subgroup analyses in the trial emulation phase. For the validation assessment, we examined the agreement in treatment effect across prognostic phenotypes using a holdout set. Lastly, a semi-synthetic data simulation was conducted to assess the bias of the trial emulation HRs and the validity of their CIs under various conditions.

#### Sensitivity analysis

In the first sensitivity analysis, we assessed the impact of different imputation strategies on GBM discriminatory performance. Multiple imputation by chained equations (MICE) was compared with median imputation for handling missing data, particularly for high-prognostic variables such as ECOG performance status and laboratory values at metastatic diagnosis, which had missing rates of 30–50%. MICE led to a marginal improvement in the time-dependent AUC for the GBM, with an average increase of 0.01 compared with median imputation (Fig. 2). Given the similar performance, median imputation was chosen for its ease of use.

**Extended Data Fig. 5:**
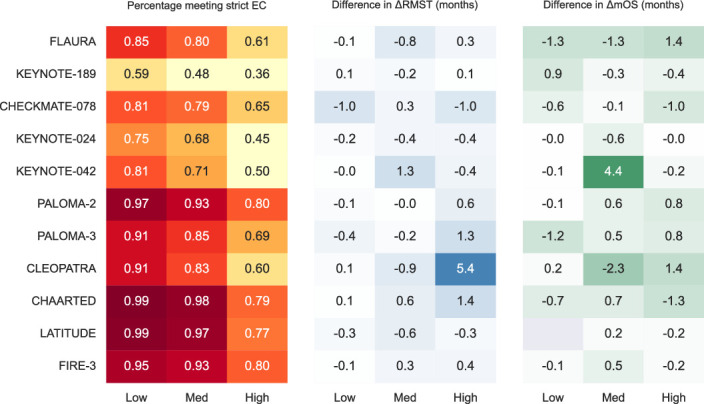
Effect of eligibility criteria strictness on trial inclusion and survival outcomes. Three heatmaps illustrating the effect of eligibility criteria (EC) on trial outcomes across prognostic phenotypes. Left heatmap shows the percentage of patients in the primary analysis meeting strict EC, which decreases as prognostic phenotype increases. Center heatmap displays the difference in ∆ restricted mean survival time (RMST) between emulated trials using key EC versus strict EC. Right heatmaps shows the difference in ∆ median overall survival (mOS) between emulated trials using key EC versus strict EC. ∆RMST and ∆mOS reflect differences in survival times between treatment and control arms. For FLAURA, KEYNOTE-024, PALOMA-2, and PALOMA-3, progression-free survival was used instead of overall survival.

In the second sensitivity analysis, we investigated whether the treatment effect differed when emulated trials were conducted using strict eligibility criteria instead of key criteria from the primary analysis. As phenotype risk increased, fewer patients met the strict eligibility criteria: more than 90% of low-risk phenotype patients met strict criteria compared with approximately 60% of high-risk phenotype patients (Extended Data Fig. 5). Despite stricter criteria, the difference in RMST between treatment and control arms remained within 3 months of the primary analysis estimate in 32 out of 33 emulated trials (11 emulated trials across three phenotypes), and the difference in mOS between treatment and control arms was within 3 months in 31 out of 33 emulated trials. This consistency suggests that stricter eligibility criteria had minimal effect on RMST or mOS.

**Extended Data Fig. 6:**
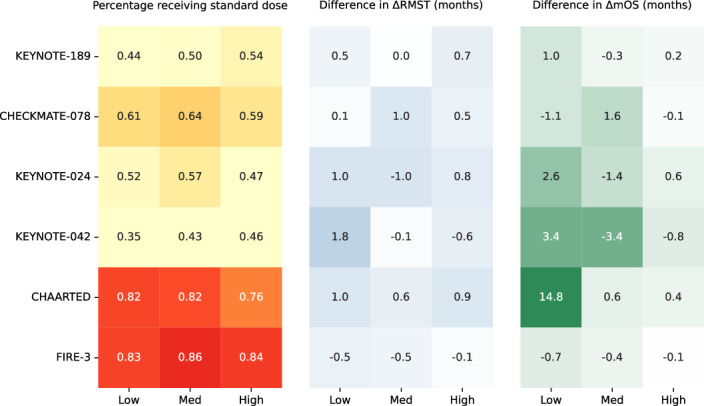
Effect of standard upfront chemotherapy dosing on survival outcomes. Three heatmaps illustrating the effect of standard upfront chemotherapy dosing, defined in Supplementary Table 31, on trial outcomes across prognostic phenotypes. Left heatmap shows the percentage of patients in the primary analysis receiving standard chemotherapy dose. Center heatmap displays the difference in ∆ restricted mean survival time (RMST) between emulated trials with standard chemotherapy dose and the primary analysis. Right heatmap shows the difference in ∆ median overall survival (mOS) between emulated trials with standard chemotherapy dose and the primary analysis. ∆RMST and ∆mOS reflect differences in survival times between treatment and control arms. For KEYNOTE-024, progression-free survival was used instead of overall survival.

In the third sensitivity analysis, we examined whether the treatment effect differed when patient inclusion depended on receiving standard doses of chemotherapeutic agents, as defined by National Comprehensive Cancer Network (NCCN) guidelines (Supplementary Table 31). This addressed concerns that high-risk phenotype patients might receive sub-therapeutic upfront doses owing to frailty. In the primary analysis, the percentage of patients receiving a standard first dose varied widely, with an average of 61% across all trials (Extended Data Fig. 6). However, this percentage did not differ significantly between prognostic phenotypes, with an average difference of less than 5%. The difference in RMST between treatment and control arms remained within 3 months of the primary analysis in 18 out of 18 emulated trials (six emulated trials across three phenotypes), and the difference in mOS between treatment and control arms was within 3 months in 15 out of 18 emulated trials. These results suggest that reduced treatment effect in high-risk phenotypes is unlikely due to sub-therapeutic upfront dosing of chemotherapeutic agents.

#### Validation analysis

In the validation analysis, we assessed treatment effect agreement across prognostic phenotypes on a holdout set in two emulated trials: KEYNOTE-189 and PALOMA-2. The GBM, trained excluding the holdout set, achieved a time-dependent AUC within 0.01 of the original GBM. HRs for the entire cohort in both the training and holdout sets were statistically significant and differed by less than 0.05 for both trials (Supplementary Tables 32 and 33). For KEYNOTE-189, statistically significant agreement in RMST difference was observed for all three phenotypes between the training and holdout sets. In PALOMA-2, agreement was achieved for two of three phenotypes. Additionally, mOS for treatment and control was within 1 month across phenotypes in the training and holdout sets for KEYNOTE-189 and typically within 3 months across phenotypes for PALOMA-2.

#### Semi-synthetic data simulation

In case 1, where both the positivity and no-unmeasured-confounder assumptions held, our method yielded low relative bias (typically less than 4%), suggesting that the HR estimates were accurate and not systematically deviated from true values. In addition, the coverage of the 95% CI was close to the nominal level of 95% (ranging from 94% to 96%) across different sample sizes and prognostic phenotypes, confirming the validity of the 95% CI for the HR (Supplementary Table 34). These results demonstrated that when both the positivity and no-unmeasured-confounder assumptions hold, the trial emulation method is robust and produces reliable, unbiased treatment effect estimates and valid inferences.

In case 2, where the positivity assumption was violated, increased bias and under-coverage of the 95% CI were observed, making estimates less reliable. More severe violations of the positivity assumption in case 3 led to even greater bias and under-coverage. In case 4, violating the no-unmeasured-confounder assumption also resulted in increased bias and under-coverage of the 95% CI. The simulations additionally showed that larger sample sizes in cases 2, 3 and 4 worsened coverage, suggesting that assumption violations are amplified in larger datasets. These findings emphasize the importance of maintaining both the positivity assumption and properly accounting for all relevant confounders to ensure reliable and unbiased estimates in trial emulations. Well-curated EHR databases have a crucial role in mitigating assumption violations by allowing for the adjustment of numerous potential confounders in the models.

### Webtool

The TrialTranslator framework is accessible as a webtool at https://www.trialtranslator.com/ and is intended for research purposes only. This tool allows users to input patient information to receive an estimated prognostic phenotype for emulated trials relevant to the patient. Based on this prognostic phenotype, the webtool then provides survival estimates for different treatments.

## Discussion

Survival times and treatment effects of anti-cancer agents reported in clinical trials are often not generalizable to many real-world patients and pose a persistent challenge for the oncology community. Our findings show that patients with low-risk or medium-risk phenotypes were more likely to experience survival times and treatment-associated survival benefits similar to those reported in RCTs. In contrast, high-risk phenotypes consistently exhibited survival times below published RCT results. In more than half of the emulated trials, high-risk phenotypes had a treatment effect, in terms of RMST or mOS difference, of less than 3 months. These findings highlight the potential of frameworks such as TrialTranslator, which integrates EHR-derived datasets, ML-based phenotyping and trial emulation, to translate oncology RCT results to individual patients. Such tools can support clinicians and patients in making informed treatment decisions, understanding expected benefits of novel therapies and planning future care.

Our results also show that high-risk phenotypes, even when meeting strict eligibility criteria, exhibited treatment effects that differed significantly from RCT benchmarks and lower-risk phenotypes. This suggests that patient prognosis, rather than eligibility criteria, better predicts survival and treatment benefit. Prospective trials should consider more sophisticated ways of evaluating patients’ prognosis upon entry rather than relying solely on strict eligibility criteria. In addition, as recommended by the American Society of Clinical Oncology and Friends of Cancer Research^34^, efforts should be made to improve the representation of high-risk subgroups in RCTs, considering that treatment effects for these individuals might differ from other participants.

These findings should be interpreted in the context of specific limitations. First, our models lacked validation on an external dataset, potentially limiting phenotyping accuracy for patients outside the Flatiron Health database. Additionally, our models calculated mortality risk scores at the time of metastatic diagnosis, which might result in phenotype misclassification for trials investigating later-line therapies (for example, CHECKMATE-078 and PALOMA-3). However, results from these later-line therapy trials were consistent with first-line therapy trials, suggesting that prognostic phenotypes remained stable throughout patients’ treatment courses.

Despite using a granular clinicogenomic dataset to account for known confounders in emulated trials, the potential for unmeasured confounders persists, possibly biasing treatment effect estimates, as our semi-synthetic data simulation demonstrated. We could not account for competing risks due to lack of cause-of-death data, although most deaths in this metastatic cohort were likely cancer related^35^. Another concern is the potential violation of the positivity assumption in the emulated trials, specifically due to the preferential use of paradigm-shifting treatments after RCT publication in high-risk groups^15,36^. Finally, crossover from the control to the treatment arm was allowed in all emulated trials, which may have attenuated the observed overall survival treatment effect, especially when the original RCTs did not permit crossover (for example, CHECKMATE-078 and KEYNOTE-042). However, most RCTs allowed crossover, and it occurred least frequently in high-risk phenotypes, where the treatment effect was already attenuated.

## Methods

### Trial selection

This study focused on aNSCLC, mBC, mPC and mCRC owing to their high prevalence in the community oncology population in the USA and globally. Our goal was to identify phase 3 RCTs related to these cancers that produced positive results and were clinically relevant to oncology practice at the time of study design in January 2023. Trials had to meet the following criteria: (1) they included only two interventional arms; (2) the treatments involved drugs or biologics recommended in the NCCN guidelines; (3) the trial protocol was accessible; and (4) at least 600 patients in the Flatiron Health database resembled the trial’s patient population (Extended Data Fig. 1).

After a review by experienced oncologists, the final selection included 11 trials spanning the four cancers: FLAURA^37^, KEYNOTE-189 (ref. ^38^), CHECKMATE-078 (ref. ^39^), KEYNOTE-024 (ref. ^40^) and KEYNOTE-042 (ref. ^41^) for aNSCLC; PALOMA-2 (ref. ^42^), PALOMA-3 (ref. ^43^) and CLEOPATRA^44^ for mBC; CHAARTED^45^ and LATITUDE^46^ for mPC; and FIRE-3 (ref. ^47^) for mCRC. Most of these trials investigated novel anti-cancer agents, including genotype-directed therapy, checkpoint inhibitors and hormone-based therapy, particularly in the first-line treatment of metastatic disease. Additional information about the trials is provided in Supplementary Table 14.

### Data source

The Flatiron Health database was used for both the prognostic model development and trial emulation steps of the study. The database included patients diagnosed with aNSCLC or mBC on or after 1 January 2011 and those diagnosed with mPC or mCRC on or after 1 January 2013. Data lock dates varied by cancer type: March 2021 for aNSCLC, September 2022 for mBC and mCRC and October 2022 for mPC.

Flatiron Health collects real-world clinical patient data from EHRs used by cancer care providers, including both community practices and academic medical centers. Most patients in the database originate from community oncology settings; relative community/academic proportions may vary depending on study cohort. By aggregating data across approximately 280 cancer clinics throughout the USA, this de-identified database offers a more comprehensive and representative view of cancer care and outcomes than EHRs from a single healthcare center.

The database includes both structured and unstructured data. Structured data, such as laboratory results and diagnosis codes, undergo harmonization and mapping to common units and terminology^48^. Unstructured data, such as clinical notes and pathology reports, are processed by oncology nurses and tumor registrars using a technology-enabled abstraction platform to extract variables such as biomarkers or date of disease progression^49^.

The accuracy of Flatiron Health’s data curation process, particularly regarding mortality data, has been rigorously validated. Patient mortality information is captured through a composite variable that integrates multiple data sources, including both structured and unstructured content from EHRs, commercial sources and the Social Security Death Index. When benchmarked against data from the National Death Index, which is the gold standard for mortality data, the sensitivity of mortality capture in the Flatiron Health database exceeded 90% across various cancer types^50^.

### Prognostic model development

The primary objective of the cancer-specific prognostic models was to accurately estimate patient survival from the time of metastatic cancer diagnosis, so that well-defined prognostic phenotypes could be generated for the emulated trial section. For each cancer type, four distinct survival-based ML models were constructed in Python version 3.7 using the scikit-survival package^51^. The first model was a gradient-boosted ensemble of regression trees using the negative log-partial likelihood of a Cox proportional hazard model as the loss function. The second model fit an ensemble of survival trees on different subsamples of the dataset, applying a log-rank split criterion. The third model was a linear survival support vector machine. Lastly, three variations of a pCox proportional hazards model were constructed: ridge ($\ell 2$ penalty), lasso (${\ell }_{1}$ penalty) and elastic net (linear combination of ${\ell }_{2}$ and ${\ell }_{1}$ penalty).

Additionally, a non-penalized Cox proportional hazards model, inspired from recent publications^26–29^, was constructed for each cancer type as a benchmark for comparison with the ML models. The selected published models were chosen based on their demonstrated discriminatory performance on externally validated datasets and their inclusion of variables available in the Flatiron Health database.

#### Data preparation

Each cancer dataset was split into two subsets: an 80% training set and a 20% test set. Given the anticipated prognostic significance of the year of metastatic diagnosis and its influence on treatment decisions, a stratified split was applied based on this variable to ensure an equitable distribution of diagnosis years across both subsets.

The index date for the models was set as the time of metastatic diagnosis. A data collection window from 90 days before to 30 days after the index date was defined, and the feature values closest to the index date were selected. The upper bound of 30 days was chosen as it represented the median start time for first-line treatment initiation. First-line treatment initiation itself was not used as an upper bound owing to the absence of documented treatment in 10–20% of patients, varying by cancer type. The end date was defined as the time of death, and patients without a recorded death date were right-censored at their last recorded activity in the EHR.

Feature categories used for prognostic model construction included demographic data, cancer characteristics, biomarkers, weight, laboratory values, medical history and medications. Medical history was identified through International Classification of Diseases (ICD) coding and mapped to the Elixhauser comorbidity list. Several features were engineered by leveraging the longitudinal nature of the dataset, such as percentage change in weight and summary laboratory measures, such as maximum, minimum and slope. Treatment data were intentionally excluded from the final feature list because the objective was to stratify patients based on mortality risk before treatment initiation. Additionally, race, ethnicity and insurance were excluded to mitigate the risk of perpetuating historical or societal biases in the predictive model^52^. For the comprehensive list of features used to construct each disease-specific model, see Supplementary Tables 15–18.

ECOG performance status and laboratory values had relatively high missing rates at the time of metastatic diagnosis, ranging from 30% to 50% depending on the cancer cohort. Special attention was given to their imputation considering their high prognostic value. The default imputation strategy involved imputing the median for laboratory values and creating a binary flag for missing values, and missing ECOG values were left as unknown. As an alternative imputation strategy, MICE was used. For each cancer type, five complete versions of the training and test sets were generated using the ‘miceforest’ Python package^53^, which uses LightGBM as the back-end model for predicting missing values. A cancer-specific survival GBM was then trained on each training set, with model performance on the test set pooled using a modified version of Rubin’s rules^54^.

#### Model evaluation

Model performance was assessed by calculating a time-dependent AUC on the test set, specifically a cumulative/dynamic AUC^55^. The AUC at time *t* is defined by equation (1), where *i* and *j* are the observed times of death for patients *i* and *j*. The function *I*(·) is an indicator function that returns 1 if a specified condition is true and 0 if the condition is false. The term *ω*_*i*_ represents the inverse probability of censoring weights for patient *i*, which adjusts for the potential bias introduced by censored data. The values $\hat{f}\left({{\bf{x}}}_{i}\right)$ and $\hat{f}\left({{\bf{x}}}_{j}\right)$ are the predicted risk scores for patients *i* and *j*, based on their respective feature vectors **x**_*i*_ and **x**_*j*_.

1 $$\widehat{\rm{AUC}}(t)=\frac{{\sum }_{i=1}^{n}{\sum }_{j=1}^{n}I(\;{y}_{j} > t)I(\;{y}_{i}\le t){\omega }_{i}I(\;{\widehat{f}}(\bf{{x}}_{j})\le{\widehat{f}}(\bf{{x}}_{i}))}{({\sum }_{j=1}^{n}I(\;{y}_{j} > t)\left)\right.)({\sum }_{i=1}^{n}I(\;{y}_{i}\le t){\omega }_{i})}$$

The numerator of the AUC formula sums over all pairs (*i*, *j*) where patient *i* has died by or at time *t* and patient *j* has survived beyond *t*, comparing their predicted risk scores and applying *ω*_*i*_ to each comparison. The denominator normalizes this sum by multiplying the number of patients who survived beyond time *t* by the weighted count of patients who died by or at time *t*. The AUC can be interpreted as the weighted probability that, given two randomly chosen patients *i* and *j*, where patient *i* dies before or at time *t* and patient *j* survives past *t*, the model assigns a risk score to patient *i* that is greater than or equal to the risk score assigned to patient *j*.

The AUC was chosen over other metrics, such as the concordance index, owing to its ability to assess a model’s discriminatory performance at clinically relevant timepoints. The AUC was calculated at 30-day intervals from the time of metastatic diagnosis up to 5 years afterwards on the test set. However, particular attention was given to the AUC at 1 year for aNSCLC and at 2 years for mBC, mCRC and mPC to determine the top-performing prognostic model. These timepoints were chosen because they align with the mOS in the Flatiron Health dataset. The 95% CIs for the AUC were bootstrapped using 10,000 iterations.

#### Hyperparameter tuning

Model hyperparameters were optimized to maximize AUC at 1 year for aNSCLC and at 2 years for mBC, mCRC and mPC. This optimization was achieved through a grid search combined with five-fold cross-validation on the training set. For ensemble models, an early stopping strategy was implemented to determine the optimal number of learners and reduce overfitting before fine-tuning the remaining hyperparameters through grid search. Detailed information on the hyperparameter search space can be found in Supplementary Table 19.

The following data transformations were implemented within the cross-validation loops: categorical variables were one-hot-encoded, and numerical variables underwent median imputation and standardization to have a mean of 0 and a standard deviation of 1.

### Trial emulation

#### Eligibility matching

In the primary analysis, we selected Flatiron Health patients who received relevant treatments at the appropriate line of therapy and exhibited correct biomarkers near the time of treatment. Biomarkers were selected up to 30 days beyond the start of treatment, accounting for the known lag time in obtaining results and their crucial role in the eligibility criteria of many trials. Treatment arms were occasionally expanded beyond those explicitly investigated in the RCT if equivalent real-world therapies were present. For instance, in PALOMA-2, which evaluated the benefit of adding palbociclib to letrozole in untreated hormone receptor-positive/HER2-negative mBC, we included real-world patients receiving any first-line aromatase inhibitor with or without palbociclib, as differences in estrogen suppression between the aromatase inhibitors are not associated with clinically significant differences in efficacy.

Two alternative eligibility matching scenarios were evaluated as part of a sensitivity analysis. The first was a strict eligibility criteria scenario, aiming to fulfill as many original trial criteria as possible within the constraints of the Flatiron Health database. Patients meeting eligibility criteria in the primary analysis were removed if they: (1) had an ICD code associated with an exclusionary comorbidity in the year before treatment start; (2) had an ECOG score exceeding the RCT cutoff, based on the score closest to and before treatment start; (3) exhibited organ dysfunction in laboratory results, defined as having any of the following within 3 months before treatment: hemoglobin < 9 g dl^−1^, creatinine > 2 mg dl^−1^ or total bilirubin > 3 mg dl^−1^; or (4) had exclusionary sites of metastases (for example, central nervous system) in the 3 months before treatment. However, certain criteria, such as measurable disease by Response Evaluation Criteria in Solid Tumors (RECIST), or specific past medical history, such as recent pregnancy or vaccinations, were not captured in the Flatiron Health database and could not be replicated. For a complete list of eligibility criteria satisfied in the emulated trials, see Supplementary Tables 20–30.

The second eligibility scenario included only patients who received a standard first dose of chemotherapeutic agents, as defined by NCCN guidelines (Supplementary Table 31). Patients with unknown first doses were excluded. Subsequent dose reduction or schedule modifications were allowed, as all RCTs permitted dose modification during the trial. Trials that included only checkpoint inhibitors, targeted therapies or hormonal therapies were not considered given the fixed-dose prescribing patterns of these agents. The CLEOPATRA trial was also not included in this subgroup analysis, despite the presence of chemotherapeutic agents, owing to low patient numbers in the primary analysis.

#### Prognostic phenotyping

Real-world patients meeting trial eligibility criteria underwent prognostic phenotyping using the top-performing model identified in step I, which was the GBM across all four cancers. The GBM generated individualized mortality risk scores for each patient, using a loss function based on the negative log-partial likelihood of a Cox proportional hazard model. This approach produces mortality risk scores analogous to the linear predictor in a Cox proportional hazards model. Patients in the emulated trials were then categorized into three prognostic phenotypes based on their mortality risk scores: high-risk (top tertile), medium-risk (middle tertile) and low-risk (bottom tertile).

#### Survival analysis

Survival analysis was conducted for the emulated trials, with time zero defined as the start of the therapy of interest. IPTW was performed to balance features between the treatment and control arms. A propensity score, representing the probability of being assigned to the treatment arm, was calculated using a logistic regression model. This model included features considered influential for selection into the treatment arm, along with prognostically significant features. Prognostic significance was determined based on the frequency of feature appearance in the GBM regression trees (Fig. 2).

The specific set of features varied by trial but typically included demographics, area-level SES, insurance status, year of metastatic diagnosis, ECOG performance status, time from initial cancer diagnosis to metastatic disease, histology, prior treatments, biomarkers, albumin, change in weight and mortality risk score estimated from the GBM. A patient’s quintile SES was determined by neighborhood factors, including income, home values, rental costs, poverty, unemployment and education level; however, this feature was available only in the breast and colorectal cancer datasets. Most feature values were selected closest to the time of metastatic diagnosis and before treatment initiation, with the exception of biomarkers, which were considered up to 30 days after treatment initiation due to potential reporting delays. To assess the balance of these features between treatment and control arms, we calculated standardized mean differences, aiming to achieve an absolute difference of less than 20% for each feature.

The propensity score, denoted as *e*_*i*_, is defined in equation (2), where *X*_*i*_ represents the features in the propensity score model for patient *i*, and *Z*_*i*_ is the indicator variable representing whether patient *i* was in the treatment arm (*Z*_*i*_ = 1) or control arm (*Z*_*i*_ = 0). During the survival analysis, patient *i* was assigned a weight, ω_*i*_, defined in equation (3).

2 $${e}_{i}=\Pr ({Z}_{i}=1|{X}_{i})$$

3 $${\omega }_{i}={Z}_{i}/{e}_{i}+(1-{Z}_{i})/(1-{e}_{i})$$

For the emulated trials consisting of the full cohort meeting strict eligibility criteria, HRs were calculated using a Cox-IPTW model. This model adjusted for features in the Cox proportional hazards regression model and balanced the distribution of features between treatment and control arms through IPTW. Robust standard errors for the HR were calculated using the Huber sandwich estimator. HRs from the emulated trials were compared with those from the RCTs by evaluating three metrics: (1) statistical significance agreement, defined as HR estimates and 95% CIs being on the same side of the null; (2) 95% CI agreement, determined by whether the emulated trial estimates fell within the 95% CI of the RCT results; and (3) standardized difference agreement, defined as an absolute standardized difference of less than 1.96 between HRs from the emulated trial and the RCT.

The treatment effect for phenotypes was estimated using the mOS and the RMST at 1 year beyond mOS from IPTW-adjusted Kaplan–Meier survival curves. The 1-year time horizon beyond mOS was selected for calculating RMST to allow adequate time for treatment and control arm separation, which is particularly relevant in the checkpoint inhibitor trials^30^. The 95% CIs for mOS, RMST, RMST difference and the Kaplan–Meier survival curves were calculated using 1,000 bootstraps, with IPTW performed within each bootstrap.

### Validation

In the validation analysis, we assessed the agreement in treatment effect across phenotypes for the KEYNOTE-189 and PALOMA-2 trials using an independent holdout set from the Flatiron Health data. These trials were selected owing to their large sample sizes, which allowed for an adequately sized holdout set. The patient population meeting trial eligibility criteria was divided into training and holdout sets, each containing half of the patients. The division was stratified by year of metastatic diagnosis and treatment receipt to ensure equitable distribution.

The GBM was trained on the full cancer cohort, excluding the holdout set. Mortality risk scores were then calculated for both the training and holdout sets. Phenotype cutoffs were determined based on tertiles from the training set patients and applied to both sets. Survival analysis was performed independently on both sets, and the results from the holdout set were compared to those from the training set to validate the consistency of treatment effect across phenotypes.

### Semi-synthetic data simulation

A semi-synthetic data simulation was performed for the KEYNOTE-189 trial to assess bias in the trial emulation HRs and evaluate the validity of resulting CIs. This simulation also analyzed how violations of key assumptions—specifically, positivity and no unmeasured confounders—impact the reliability of the HR estimates. The KEYNOTE-189 trial was selected owing to its large sample size and the lower likelihood of proportional hazards violations within phenotypes, as both treatment and control arms included chemotherapy as part of the interventions.

The semi-synthetic dataset was generated using a two-step procedure. First, a logistic regression model simulated treatment assignments based on patient features from the Flatiron Health database. The features selected were the same as those used in the KEYNOTE-189 propensity model in the primary analysis. Mean imputation was performed for missing values. Second, a Cox model was used to simulate survival time and death. Survival times were generated using an exponential distribution, where the rate parameter was the product of the baseline hazard and the individual hazard obtained from the Cox model. Event status was simulated using a binomial distribution, with the probability of death based on the mortality risk estimates from the Cox model, ensuring that the semi-synthetic dataset had similar hazard and survival characteristics to the original data.

Four cases were assessed. In case 1, both the positivity and no-unmeasured-confounder assumptions were maintained. In case 2, the positivity assumption was violated, with 30% of patients randomly chosen not to receive treatment by setting their propensity score to 0 while maintaining the no-unmeasured-confounder assumption. Case 3 involved a more severe positivity violation, with 40% of patients never receiving treatment. In case 4, the positivity assumption was maintained, but the no-unmeasured-confounder assumption was violated by excluding variables such as weight percentage change at the time of advanced diagnosis and PD-L1 status from the propensity score estimation. These variables were specifically selected because they are often missing at treatment initiation in real-world databases. Of note, these variables were included in our trial emulations.

For each case, samples of 10,000, 15,000 or 20,000 patients from the semi-synthetic dataset were analyzed. A Cox-IPTW model was fitted to calculate the HR for the entire dataset and for each prognostic phenotype, with the model specifics varying by case. This process was repeated 2,000 times for each sample size. Bias was calculated as the difference between the estimated HR from the semi-synthetic dataset and the true HR, and average bias was computed across the 2,000 iterations. The coverage rate was calculated as the proportion of true HRs that fell within the 95% CIs of the estimated HRs across the 2,000 iterations.

### Reporting summary

Further information on research design is available in the Nature Portfolio Reporting Summary linked to this article.

## Online content

Any methods, additional references, Nature Portfolio reporting summaries, source data, extended data, supplementary information, acknowledgements, peer review information; details of author contributions and competing interests; and statements of data and code availability are available at 10.1038/s41591-024-03352-5.

## Supplementary information

Supplementary Tables 1–34

Reporting Summary

## Figures and Tables

**Extended Data Table 1:**
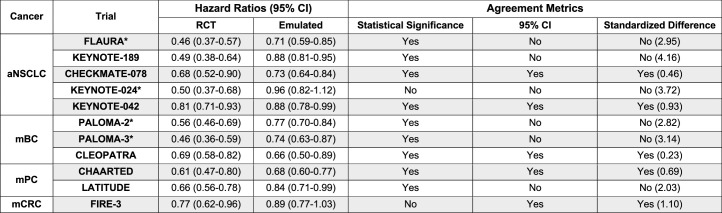
HRs and agreement metrics for emulated trials involving the full cohort

HRs for overall survival with treatment in the emulated trials involving the full cohort using strict eligibility criteria. Asterisks (*) indicate progression-free survival HRs. Agreement metrics compare emulated trials to respective RCTs: (1) statistical significance: HR estimates and 95% CI on the same side of null; (2) 95% CI agreement: emulated HR within the RCT’s 95% CI; and (3) standardized difference: absolute difference between emulated and RCT HRs is less than 1.96.

**Extended Data Table 2:**
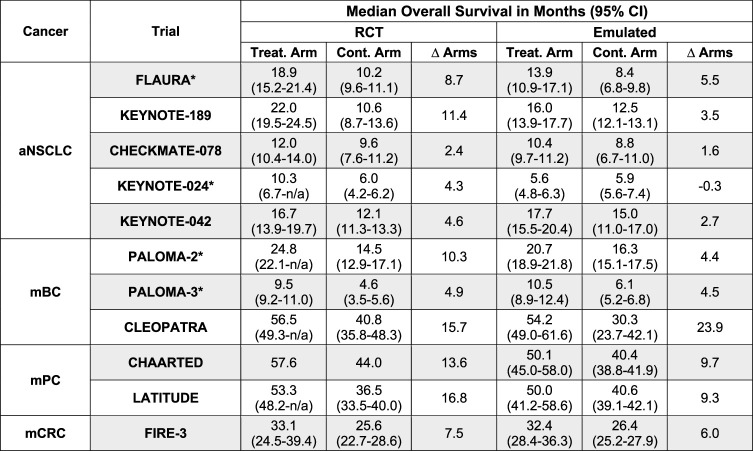
mOS times for emulated trials involving the full cohort

mOS in months for treatment (Treat.) and control (Cont.) arms for a variety of emulated trials involving the entire cohort using strict eligibility criteria. Asterisks (*) indicate mPFS. Δ Arms denotes difference in survival time between treatment and control arms. Note: CHAARTED results for the RCT did not publish CIs.

**Extended Data Table 3:**
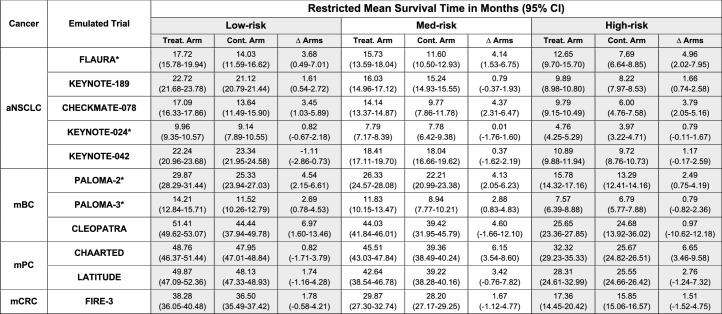
RMSTs across prognostic phenotypes

RMST comparison in months between treatment (Treat.) and control (Cont.) arms for a variety of emulated trials, stratified by prognostic phenotypes and involving patients with aNSCLC, mBC, mPC or mCRC. RMST was calculated at 1.5 years for KEYNOTE-024; at 2 years for PALOMA-3; at 3 years for FLAURA, KEYNOTE-189, CHECKMATE-078 and KEYNOTE-042; at 4 years for PALOMA-2 and FIRE-3; and at 5 years for CLEOPATRA, CHAARTED and LATITUDE. These timepoints correspond to approximately 1 year beyond the mOS or mPFS for the respective RCT. For trials marked with an asterisk (*), restricted mPFS time is reported instead. Δ Arms denotes difference survival time between treatment and control arms.

## Data Availability

The Flatiron Health data used in this study were made available as part of an academic collaboration with the University of Pennsylvania. The authors had full access to the data and were responsible for conducting the analyses. This research was conducted in compliance with all relevant ethical regulations. Institutional review board approval from the University of Pennsylvania, including a waiver of informed consent, was obtained before the study was conducted. These de-identified data may be made available upon reasonable request via a proposal-based process. Interested researchers can contact DataAccess@flatiron.com.

## Code availability

The open-source Python code for TrialTranslator and the associated webtool are available on GitHub (https://github.com/xavier-orcutt/TrialTranslator-notebooks and https://github.com/xavier-orcutt/TrialTranslator-webtool).
