# Supplementary material for: Evaluating generalizability of oncology trial results to real-world patients using machine learning-based trial emulations
Source: Nat Med. 2025 Jan 3;31(2):457–65. doi: 10.1038/s41591-024-03352-5 (PMC11835724; doi:10.1038/s41591-024-03352-5)
Supplement: Supplementary file 1 — Supplementary Tables 1–34 [file 41591_2024_3352_MOESM1_ESM.pdf]

nature medicine

Supplementary information https://doi.org/10.1038/s41591-024-03352-5

Evaluating generalizability of oncology trial results to real-world patients using machine learning-based trial emulations

In the format provided by the authors and unedited

TABLE OF CONTENTS PAGE

Supplementary Table 1 2

Supplementary Tables 2-12 3-13

Supplementary Table 13 14

Supplementary Table 14 15

Supplementary Tables 15-18 16-27

Supplementary Table 19 28

Supplementary Tables 20-30 29-42

Supplementary Table 31 43

Supplementary Table 32 44

Supplementary Table 33 45

Supplementary Table 34 46

aNSCLC

Model	1-yr AUC on test set (95% CI)	1-yr AUC on training set (95% CI)
GBM, MICE	0.789 (0.784-0.795)	0.807 (0.794-0.819)
GBM	0.783 (0.775-0.791)	0.788 (0.784-0.792)
RSF	0.773 (0.765-0.781)	0.873 (0.870-0.877)
pCox, ridge	0.748 (0.738-0.755)	0.751 (0.747-0.755)
SVM	0.741 (0.732-0.750)	0.747 (0.742-0.751)
pCox, lasso	0.734 (0.725-0.743)	0.735 (0.731-0.740)
pCox, elastic net	0.733 (0.724-0.742)	0.735 (0.731-0.739)
Cox	0.698 (0.684-0.713)	0.700 (0.693-0.707)

mBC

Model	2-yr AUC on test set (95% CI)	2-yr AUC on training set (95% CI)
GBM, MICE	0.818 (0.812-0.824)	0.841 (0.830-0.852)
GBM	0.814 (0.802-0.826)	0.850 (0.845-0.856)
RSF	0.795 (0.783-0.808)	0.883 (0.879-0.888)
SVM	0.785 (0.773-0.798)	0.790 (0.784-0.797)
pCox, lasso	0.783 (0.770-0.796)	0.786 (0.779-0.792)
pCox, elastic net	0.783 (0.770-0.796)	0.786 (0.779-0.792)
pCox, ridge	0.783 (0.770-0.796)	0.785 (0.778-0.791)
Cox	0.754 (0.733-0.776)	0.755 (0.744-0.766)

mPC

Model	2-yr AUC on test set (95% CI)	2-yr AUC on training set (95% CI)
GBM, MICE	0.772 (0.764-0.781)	0.823 (0.801-0.846)
GBM	0.754 (0.736-0.773)	0.785 (0.776-0.794)
RSF	0.747 (0.728-0.766)	0.836 (0.828-0.843)
SVM	0.741 (0.722-0.760)	0.746 (0.737-0.756)
pCox, ridge	0.739 (0.721-0.758)	0.743 (0.734-0.753)
pCox, elastic net	0.731 (0.712-0.750)	0.735 (0.725-0.745)
pCox, lasso	0.730 (0.710-0.749)	0.734 (0.724-0.744)
Cox	0.612 (0.582-0.642)	0.625 (0.611-0.641)

mCRC

Model	2-yr AUC on test set (95% CI)	2-yr AUC on training set (95% CI)
GBM, MICE	0.778 (0.771-0.785)	0.831 (0.807-0.856)
GBM	0.768 (0.755-0.780)	0.817 (0.812-0.823)
RSF	0.743 (0.730-0.757)	0.879 (0.874-0.883)
SVM	0.738 (0.724-0.751)	0.736 (0.730-0.743)
pCox, ridge	0.736 (0.722-0.749)	0.732 (0.725-0.739)
pCox, lasso	0.721 (0.707-0.735)	0.712 (0.705-0.719)
pCox, elastic net	0.721 (0.707-0.734)	0.712 (0.705-0.719)
Cox	0.641 (0.627-0.656)	0.646 (0.638-0.653)

Supplementary Table 1: Performance of models on test and training sets in patients with advanced non-small cell lung cancer (aNSCLC), metastatic breast cancer (mBC), metastatic prostate cancer (mPC), or metastatic colorectal cancer (mCRC). Key abbreviations are as follows: AUC (Area Under the Curve), GBM (Gradient Boosting Model), MICE (Multiple Imputation by Chained Equations), pCOX (Penalized Cox), RSF (Random Survival Forest), and SVM (Support Vector Machines).

FLAURA		Low	Med	High	RCT
**n**		1207	1207	1207	556
**Age at metastatic diagnosis, median**		65	72	75	64
**Male sex, n (%)**		321 (26.6)	410 (34.0)	522 (43.2)	206 (37)
**Race, n (%)**	*White*	663 (54.9)	722 (59.8)	724 (60.0)	201 (36)
	Black	79 (6.5)	101 (8.4)	85 (7.0)	
	Asian	194 (16.1)	128 (10.6)	114 (9.4)	347 (62)
	Other	140 (11.6)	121 (10.0)	135 (11.2)	8 (1)
**ECOG, n (%)**	*<2*	468 (38.8)	383 (31.7)	357 (29.6)	
	≥2	11 (0.9)	44 (3.6)	234 (19.4)	0 (0)
	Unknown	728 (60.3)	780 (64.6)	616 (51.0)	
**History of smoking, n (%)**		404 (33.5)	682 (56.5)	826 (68.4)	
**Histology, n (%)**	*Non-squamous*	1178 (97.6)	1112 (92.1)	1044 (86.5)	547 (98)
	Squamous	15 (1.2)	64 (5.3)	104 (8.6)	
	NOS	14 (1.2)	31 (2.6)	59 (4.9)	
**Year of metastatic diagnosis, n (%)**	*2011-2014*	420 (34.8)	523 (43.3)	555 (46.0)	
	2015-2018	489 (40.5)	446 (37.0)	428 (35.5)	
	2019-2022	298 (24.7)	238 (19.7)	224 (18.6)	0 (0)
**Brain metastasis, n (%)**		95 (7.9)	113 (9.4)	133 (11.0)	116 (21)
**Labs at start of treatment, n (%)**	*Creatinine >2 mg/dl*	3 (0.4)	6 (0.7)	25 (2.6)	0 (0)
	Hemoglobin <9 g/dl	5 (0.6)	13 (1.6)	35 (3.6)	0 (0)
	Total bilirubin >3 mg/dl	0 (0)	1 (0.1)	2 (0.2)	0 (0)
**Time to death from diagnosis (days), median**		744	569	258.5	

Supplementary Table 2: Baseline characteristics of the patients in the emulated FLAURA trial from the primary analysis, stratified by risk phenotype. The RCT column displays the baseline values of participants reported in Table 1 of the published trial.

KEYNOTE-189		Low	Med	High	RCT
n		9680	9680	9680	616
Age at metastatic diagnosis, median		66	69	70	64.4
Male sex, n (%)		4546 (47.0)	5574 (57.6)	6312 (65.2)	363 (59)
Race	*White*	6727 (69.5)	6824 (70.5)	6714 (69.4)	
	Black	930 (9.6)	829 (8.6)	822 (8.5)	
	Asian	169 (1.7)	134 (1.4)	99 (1.0)	
	Other	944 (9.8)	943 (9.7)	896 (9.3)	
ECOG, n (%)	*<2*	4591 (47.4)	4454 (46.0)	3713 (38.4)	612 (99)
	≥2	142 (1.5)	592 (6.1)	1984 (20.5)	1 (1)
	Unknown	4947 (51.1)	4634 (47.9)	3983 (41.1)	
History of smoking, n (%)		8712 (90.0)	9041 (93.4)	9122 (94.2)	543 (88)
Histology, n (%)	*Non-squamous*	6881 (71.1)	6268 (64.8)	5975 (61.7)	592 (96)
	Squamous	2491 (25.7)	2933 (30.3)	2947 (30.4)	10 (2)
	NOS	308 (3.2)	479 (4.9)	758 (7.8)	14 (2)
Year of metastatic diagnosis, n (%)	*2011-2014*	2999 (31.0)	3307 (34.2)	3362 (34.7)	
	2015-2018	4585 (47.4)	4474 (46.2)	4419 (45.7)	
	2019-2022	2096 (21.7)	1899 (19.6)	1899 (19.6)	0 (0)
Brain metastasis, n (%)		378 (3.9)	704 (7.3)	879 (9.1)	108 (17)
PDL1, n (%)	*0%*	1174 (12.1)	1154 (11.9)	1221 (12.6)	190 (31)
	1-49%	1073 (11.1)	1058 (10.9)	924 (9.5)	186 (30)
	50-100%	864 (8.9)	598 (6.2)	550 (5.7)	202 (33)
	Unknown	6569 (67.9)	6870 (71.0)	6985 (72.2)	
Labs at start of treatment, n (%)	*Creatinine >2 mg/dl*	47 (0.5)	122 (1.3)	163 (1.8)	0 (0)
	Hemoglobin <9 g/dl	157 (1.7)	251 (2.7)	580 (6.2)	0 (0)
	Total bilirubin >3 mg/dl	1 (0.0)	2 (0.0)	35 (0.4)	0 (0)
Time to death from diagnosis (days), median		453	299	153	

Supplementary Table 3: Baseline characteristics of the patients in the emulated KEYNOTE-189 trial from the primary analysis, stratified by risk phenotype. The RCT column displays the baseline values of participants reported in Table 1 of the published trial.

CHECKMATE-078		Low	Med	High	RCT
**n**		1358	1357	1358	504
**Age at metastatic diagnosis, median**		65	69	69	60
**Male sex, n (%)**		621 (45.7)	795 (58.6)	879 (64.7)	397 (79)
**Race**	*White*	952 (70.1)	962 (70.9)	962 (70.8)	
	Black	142 (10.5)	153 (11.3)	149 (11.0)	
	Asian	20 (1.5)	17 (1.3)	12 (0.9)	
	Other	133 (9.8)	132 (9.7)	115 (8.5)	
**ECOG, n (%)**	*<2*	639 (47.1)	644 (47.5)	596 (43.9)	503 (99)
	≥2	20 (1.5)	71 (5.2)	241 (17.7)	0
	Unknown	699 (51.5)	642 (47.3)	521 (38.4)	1 (1)
**History of smoking, n (%)**		1184 (87.2)	1280 (94.3)	1290 (95.0)	354 (70)
**Histology, n (%)**	*Non-squamous*	979 (72.1)	869 (64.0)	776 (57.1)	304 (60)
	Squamous	346 (25.5)	447 (32.9)	505 (37.2)	200 (40)
	NOS	33 (2.4)	41 (3.0)	77 (5.7)	0 (0)
**Year of metastatic diagnosis, n (%)**	*2011-2014*	371 (27.3)	322 (23.7)	277 (20.4)	
	2015-2018	950 (70.0)	995 (73.3)	1039 (76.5)	
	2019-2022	37 (2.7)	40 (2.9)	42 (3.1)	0 (0)
**Brain metastasis, n (%)**		47 (3.5)	109 (8.0)	127 (9.4)	72 (14)
**PDL1, n (%)**	*0%*	182 (13.4)	180 (13.3)	186 (13.7)	205 (41)
	1-49%	116 (8.5)	126 (9.3)	117 (8.6)	252 (50)
	50-100%	57 (4.2)	44 (3.2)	38 (2.8)	
	Unknown	1207 (49.7)	1366 (56.3)	1463 (60.2)	47 (9)
**Labs at start of treatment, n (%)**	*Creatinine >2 mg/dl*	21 (1.6)	26 (1.9)	32 (2.4)	0 (0)
	Hemoglobin <9 g/dl	67 (5.0)	114 (8.5)	181 (13.4)	0 (0)
	Total bilirubin >3 mg/dl	1 (0.1)	1 (0.1)	3 (0.2)	0 (0)
**Time to death from diagnosis (days), median**		623.5	465	319	

Supplementary Table 4: Baseline characteristics of the patients in the emulated CHECKMATE-078 trial from the primary analysis, stratified by risk phenotype. The RCT column displays the baseline values of participants reported in Table 1 of the published trial.

KEYNOTE-024		Low	Med	High	RCT
**n**		707	706	707	305
**Age at metastatic diagnosis, median**		66	73	73	65
**Male sex, n (%)**		325 (46.0)	362 (51.3)	442 (62.5)	187 (61)
**Race**	*White*	473 (66.9)	477 (67.6)	486 (68.7)	
	Black	62 (8.8)	52 (7.4)	62 (8.8)	
	Asian	18 (2.5)	12 (1.7)	12 (1.7)	
	Other	62 (8.8)	67 (9.5)	80 (11.3)	
**ECOG, n (%)**	*<2*	371 (52.5)	375 (53.1)	276 (39.0)	304 (99)
	≥2	15 (2.1)	58 (8.2)	218 (30.8)	
	Unknown	321 (45.4)	273 (38.7)	213 (30.1)	
**History of smoking, n (%)**		638 (90.2)	656 (92.9)	672 (95.0)	281 (92)
**Histology, n (%)**	*Non-squamous*	565 (79.9)	503 (71.2)	460 (65.1)	249 (82)
	Squamous	121 (17.1)	167 (23.7)	203 (28.7)	66 (22)
	NOS	21 (3.0)	36 (5.1)	44 (6.2)	
**Year of metastatic diagnosis, n (%)**	*2011-2014*	0 (0)	2 (0.3)	1 (0.1)	
	2015-2018	479 (67.8)	473 (67.0)	452 (63.9)	
	2019-2022	228 (32.2)	231 (32.7)	254 (35.9)	0 (0)
**Brain metastasis, n (%)**		84 (11.1)	77 (10.2)	86 (11.4)	28 (9)
**PDL1, n (%)**	*100%*	89 (12.6)	98 (13.9)	90 (12.7)	
	90-99%	216 (30.6)	222 (31.4)	219 (31.0)	
	80-89%	104 (14.7)	100 (14.2)	116 (16.4)	
	70-79%	106 (15.0)	103 (14.6)	96 (13.6)	
	60-69%	88 (12.4)	84 (11.9)	76 (10.7)	
	50-59%	104 (14.7)	99 (14.0)	110 (15.6)	
**Labs at start of treatment, n (%)**	*Creatinine >2 mg/dl*	4 (0.6)	11 (1.7)	15 (2.2)	0 (0)
	Hemoglobin <9 g/dl	11 (1.7)	25 (3.8)	61 (8.9)	0 (0)
	Total bilirubin >3 mg/dl	1 (0.2)	0 (0)	5 (0.7)	0 (0)
**Time to death from diagnosis (days), median**		354	259.5	109	

Supplementary Table 5: Baseline characteristics of the patients in the emulated KEYNOTE-024 trial from the primary analysis, stratified by risk phenotype. The RCT column displays the baseline values of participants reported in Table 1 of the published trial.

KEYNOTE-042		Low	Med	High	RCT
**n**		1849	1849	1850	1274
**Age at metastatic diagnosis, median**		67	72	73	63
**Male sex, n (%)**		830 (44.9)	976 (52.8)	1122 (60.6)	902 (71)
**Race**	*White*	1226 (66.3)	1295 (70.0)	1263 (68.3)	
	Black	180 (9.7)	143 (7.7)	170 (9.2)	
	Asian	32 (1.7)	30 (1.6)	29 (1.6)	
	Other	181 (9.8)	170 (9.2)	193 (10.4)	
**ECOG, n (%)**	*<2*	1012 (54.7)	932 (50.4)	782 (42.3)	1274 (100)
	≥2	38 (2.1)	169 (9.1)	502 (27.1)	0 (0)
	Unknown	799 (43.2)	748 (40.5)	566 (30.6)	0 (0)
**History of smoking, n (%)**		1683 (91.0)	1753 (94.8)	1764 (95.4)	992 (78)
**Histology, n (%)**	*Non-squamous*	1360 (73.6)	1176 (63.6)	1102 (59.6)	782 (61)
	Squamous	446 (24.1)	588 (31.8)	633 (34.2)	492 (39)
	NOS	43 (2.3)	85 (4.6)	115 (6.2)	
**Year of metastatic diagnosis, n (%)**	*2011-2014*	21 (1.1)	13 (0.7)	4 (0.2)	
	2015-2018	1159 (62.7)	1195 (64.6)	1175 (63.5)	
	2019-2022	669 (36.2)	641 (34.7)	671 (36.3)	0 (0)
**Brain metastasis, n (%)**		99 (5.4)	156 (8.4)	159 (8.6)	70 (5)
**PDL1, n (%)**	*1-49%*	645 (34.9)	737 (39.9)	737 (39.8)	675 (53)
	≥50%	1204 (65.1)	1112 (60.1)	1113 (60.2)	599 (47)
**Labs at start of treatment, n (%)**	*Creatinine >2 mg/dl*	10 (0.6)	30 (1.7)	43 (2.4)	0 (0)
	Hemoglobin <9 g/dl	33 (1.9)	69 (3.9)	157 (8.8)	0 (0)
	Total bilirubin >3 mg/dl	0 (0)	2 (0.1)	7 (0.4)	0 (0)
**Time to death from diagnosis (days), median**		417.5	296.5	142	

Supplementary Table 6: Baseline characteristics of the patients in the emulated KEYNOTE-042 trial from the primary analysis, stratified by risk phenotype. The RCT column displays the baseline values of participants reported in Table 1 of the published trial.

PALOMA-2		Low	Med	High	RCT
**n**		1578	1578	1578	666
**Age at metastatic diagnosis, median**		64	69	73	62
**Race**	*White*	1124 (71.2)	1084 (68.7)	1094 (69.3)	516 (77)
	Black	117 (7.4)	124 (7.9)	166 (10.5)	11 (2)
	Asian	35 (2.2)	31 (2.0)	23 (1.5)	95 (14)
	Other	173 (11.0)	186 (11.8)	149 (9.4)	44 (7)
**ECOG, n (%)**	*<2*	674 (42.7)	621 (39.4)	611 (38.7)	654 (98)
	≥2	33 (2.1)	95 (6.0)	321 (20.3)	12 (2)
	Unknown	871 (55.2)	862 (54.6)	646 (40.9)	0 (0)
**Year of metastatic diagnosis, n (%)**	*2011-2014*	405 (25.7)	451 (28.6)	341 (21.6)	
	2015-2018	664 (42.1)	620 (39.3)	618 (39.2)	
	2019-2022	509 (32.3)	507 (32.1)	619 (39.2)	0 (0)
**Metastasis free interval, n (%)**	*De novo*	714 (45.2)	746 (47.3)	737 (46.7)	248 (37)
	≤12 months	20 (1.3)	46 (2.9)	59 (3.7)	147 (22)
	>12 months	844 (53.5)	786 (49.8)	782 (49.6)	271 (41)
**Metastatic disease site, n (%)**	*Visceral*	541 (34.3)	704 (44.6)	950 (60.2)	324 (49)
	Non-visceral	1027 (65.1)	865 (54.8)	623 (39.5)	342 (51)
	Unknown	10 (0.6)	9 (0.6)	5 (0.3)	0 (0)
**Labs at start of treatment, n (%)**	*Creatinine >2 mg/dl*	5 (0.4)	6 (0.6)	24 (2.1)	0 (0)
	Hemoglobin <9 g/dl	15 (1.3)	32 (2.9)	78 (6.9)	0 (0)
	Total bilirubin >3 mg/dl	0 (0)	0 (0)	5 (0.4)	0 (0)
**Time to death from diagnosis (days), median**		1316	1016	517	

Supplementary Table 7: Baseline characteristics of the patients in the emulated PALOMA-2 trial from the primary analysis, stratified by risk phenotype. The RCT column displays the baseline values of participants reported in Table 1 of the published trial.

PALOMA-3		Low	Med	High	RCT
**n**		385	383	385	521
**Age at metastatic diagnosis, median**		65	68	74	
**Race**	*White*	284 (73.8)	294 (76.8)	276 (71.7)	385 (74)
	Black	33 (8.6)	33 (8.6)	40 (10.4)	29 (6)
	Asian	5 (1.3)	4 (1.0)	9 (2.3)	105 (20)
	Other	45 (11.7)	35 (9.1)	41 (10.6)	0 (0)
**ECOG, n (%)**	*<2*	120 (31.2)	136 (35.5)	161 (41.8)	521 (100)
	≥2	5 (1.3)	24 (6.3)	51 (13.2)	
	Unknown	260 (67.5)	223 (58.2)	173 (44.9)	
**Year of metastatic diagnosis, n (%)**	*2011-2014*	229 (59.5)	197 (51.4)	124 (32.2)	
	2015-2018	128 (33.2)	141 (36.8)	161 (41.8)	
	2019-2022	28 (7.3)	45 (11.7)	100 (26.0)	0 (0)
**Metastasis free interval, n (%)**	*≤24 months*	7 (1.8)	37 (9.7)	77 (20.0)	62 (12)
	>24 months	246 (63.9)	234 (61.1)	227 (59.0)	292 (56)
**Metastatic disease site, n (%)**	*Visceral*	125 (32.5)	183 (47.8)	230 (59.7)	311 (60)
	Non-visceral	256 (66.5)	198 (51.7)	153 (39.7)	210 (40)
	Unknown	4 (1.0)	2 (0.5)	2 (0.5)	0 (0)
**Labs at start of treatment, n (%)**	*Creatinine >2 mg/dl*	6 (1.7)	3 (0.9)	5 (1.4)	0 (0)
	Hemoglobin <9 g/dl	6 (1.7)	5 (1.4)	25 (7.1)	0 (0)
	Total bilirubin >3 mg/dl	0 (0)	0 (0)	1 (0.3)	0 (0)
**Time to death from diagnosis (days), median**		1669	1128	571	

Supplementary Table 8: Baseline characteristics of the patients in the emulated PALOMA-3 trial from the primary analysis, stratified by risk phenotype. The RCT column displays the baseline values of participants reported in Table 1 of the published trial.

CLEOPATRA		Low	Med	High	RCT
**n**		431	431	432	808
**Age at metastatic diagnosis, median**		56	60	66	
**Race**	*White*	255 (59.2)	269 (62.4)	286 (66.2)	480 (59)
	Black	66 (15.3)	57 (13.2)	59 (13.7)	30 (4)
	Asian	14 (3.2)	12 (2.8)	5 (1.2)	261 (32)
	Other	59 (13.7)	55 (12.8)	42 (9.7)	37 (5)
**ECOG, n (%)**	*<2*	222 (51.5)	216 (50.1)	218 (50.5)	
	≥2	3 (0.7)	20 (4.6)	57 (13.2)	
	Unknown	206 (47.8)	195 (45.2)	157 (36.3)	
**Year of metastatic diagnosis, n (%)**	*2011-2014*	108 (25.1)	118 (27.4)	94 (21.8)	
	2015-2018	194 (45.0)	196 (45.5)	205 (47.5)	
	2019-2022	129 (29.9)	117 (27.1)	133 (30.8)	0 (0)
**Metastasis free interval, n (%)**	*De novo*	317 (73.5)	213 (49.4)	183 (42.4)	
	≤12 months	4 (0.9)	10 (2.3)	22 (5.1)	
	>12 months	110 (25.5)	208 (48.3)	227 (52.5)	
**Metastatic disease site, n (%)**	*Visceral*	257 (59.6)	320 (74.2)	373 (86.3)	630 (78)
	Non-visceral	173 (40.1)	109 (25.3)	58 (13.4)	178 (22)
	Unknown	1 (0.2)	2 (0.5)	1 (0.2)	0 (0)
**ER or PR positive**		290 (67.3)	266 (61.7)	214 (49.5)	388 (48)
**Labs at start of treatment, n (%)**	*Creatinine >2 mg/dl*	1 (0.2)	6 (1.5)	8 (2.0)	0 (0)
	Hemoglobin <9 g/dl	8 (2.0)	5 (1.3)	24 (5.9)	0 (0)
	Total bilirubin >3 mg/dl	1 (0.2)	4 (1.0)	17 (4.2)	0 (0)
**Time to death from diagnosis (days), median**		1094	934	408	

Supplementary Table 9: Baseline characteristics of the patients in the emulated CLEOPATRA trial from the primary analysis, stratified by risk phenotype. The RCT column displays the baseline values of participants reported in Table 1 of the published trial.

CHAARTED		Low	Med	High	RCT
**n**		2466	2465	2466	790
**Age at metastatic diagnosis, median**		67	74	78	64
**Race**	*White*	1555 (63.1)	1482 (60.1)	1526 (61.9)	377 (48)
	Black	228 (9.2)	256 (10.4)	247 (10.0)	76 (10)
	Asian	31 (1.3)	42 (1.7)	33 (1.3)	
	Other	426 (17.3)	403 (16.3)	376 (15.2)	10 (1)
**ECOG, n (%)**	*<2*	585 (23.7)	560 (22.7)	733 (29.7)	778 (98)
	≥2	8 (0.3)	37 (1.5)	418 (17.0)	12 (2)
	Unknown	1873 (76.0)	1868 (75.8)	1315 (53.3)	
**Year of metastatic diagnosis, n (%)**	*2011-2014*	501 (20.3)	582 (23.6)	386 (15.7)	
	2015-2018	1245 (50.5)	1168 (47.4)	1149 (46.6)	
	2019-2022	720 (29.2)	715 (29.0)	931 (37.8)	0 (0)
**Gleason score at first diagnosis, n (%)**	*4-6*	112 (4.5)	124 (5.0)	84 (3.4)	42 (5)
	7	576 (23.4)	279 (11.3)	124 (5.0)	179 (23)
	8-10	1473 (59.7)	1275 (51.7)	820 (33.3)	484 (61)
	Unknown	305 (12.4)	787 (31.9)	1438 (58.3)	85 (11)
**Metastatic disease site, n (%)**	*Visceral*	66 (2.7)	76 (3.1)	140 (5.7)	123 (16)
	Non-visceral	491 (19.9)	668 (27.1)	1049 (42.5)	
	Unknown	1909 (77.4)	1721 (69.8)	1277 (51.8)	
**Prior therapy, n (%)**	*No local therapy*	1582 (64.2)	1901 (77.1)	2038 (82.6)	575 (73)
	Prostatectomy	574 (23.3)	183 (7.4)	136 (5.5)	154 (19)
	Radiation	297 (12.0)	370 (15.0)	278 (11.3)	60 (8)
	Other	13 (0.5)	11 (0.4)	14 (0.6)	
**PSA (ng/ml) at metastatic diagnosis, median**		29.7	93.4	188	51.5
**Labs at start of treatment, n (%)**	*Creatinine >2 mg/dl*	12 (1.5)	22 (2.9)	109 (7.6)	0 (0)
	Hemoglobin <9 g/dl	8 (1.0)	27 (3.6)	207 (14.5)	0 (0)
	Total bilirubin >3 mg/dl	0 (0)	1 (0.1)	3 (0.2)	0 (0)
**Time to death from diagnosis (days), median**		1072	889	542	

Supplementary Table 10: Baseline characteristics of the patients in the emulated CHAARTED trial from the primary analysis, stratified by risk phenotype. The RCT column displays the baseline values of participants reported in Table 1 of the published trial.

LATITUDE		Low	Med	High	RCT
n		2348	2347	2348	1199
Age at metastatic diagnosis, median		67.5	76	79	68
Race	*White*	1472 (62.7)	1413 (60.2)	1444 (61.5)	
	Black	221 (9.4)	230 (9.8)	235 (10.0)	
	Asian	33 (1.4)	45 (1.9)	30 (1.3)	
	Other	394 (16.8)	381 (16.2)	346 (14.7)	
ECOG, n (%)	*<2*	533 (22.7)	508 (21.6)	681 (29.0)	
	≥2	7 (0.3)	36 (1.5)	413 (17.6)	
	Unknown	1808 (77.0)	1803 (76.8)	1254 (53.4)	
Year of metastatic diagnosis, n (%)	*2011-2014*	469 (20.0)	561 (23.9)	352 (15.0)	
	2015-2018	1112 (47.4)	1032 (44.0)	995 (42.4)	
	2019-2022	767 (32.7)	754 (32.1)	1001 (42.6)	0 (0)
Gleason score at first diagnosis, n (%)	*4-6*	109 (4.6)	126 (5.4)	78 (3.3)	5 (<1)
	7	587 (25.0)	272 (11.6)	125 (5.3)	24 (2)
	8-10	1377 (58.6)	1217 (51.9)	739 (31.5)	1170 (98)
	Unknown	275 (11.7)	732 (31.2)	1406 (59.9)	
Metastatic disease site, n (%)	*Visceral*	59 (2.5)	66 (2.8)	128 (5.5)	
	Non-visceral	428 (18.2)	581 (24.8)	981 (41.8)	
	Unknown	1861 (79.3)	1700 (72.4)	1239 (52.8)	
Prior therapy, n (%)	*Prostatectomy*	606 (25.8)	184 (7.8)	126 (5.4)	
	Radiation	301 (12.8)	386 (16.4)	271 (11.5)	
	Other	15 (0.6)	10 (0.4)	13 (0.6)	
	Unknown	1426 (60.7)	1767 (75.3)	1938 (82.5)	
PSA (ng/ml) at metastatic diagnosis, median		24.1	76.2	196	
Labs at start of treatment, n (%)	*Creatinine >2 mg/dl*	11 (1.4)	22 (3.2)	107 (7.9)	0 (0)
	Hemoglobin <9 g/dl	7 (0.9)	21 (3.0)	207 (15.3)	0 (0)
	Total bilirubin >3 mg/dl	0 (0)	1 (0.1)	3 (0.2)	0 (0)
Time to death from diagnosis (days), median		1048	847	509	

Supplementary Table 11: Baseline characteristics of the patients in the emulated LATITUDE trial from the primary analysis, stratified by risk phenotype. The RCT column displays the baseline values of participants reported in Table 1 of the published trial.

FIRE-3		Low	Med	High	RCT
**n**		1214	1213	1214	407
**Age at metastatic diagnosis, median**		57	64	67	64
**Male sex, n (%)**		680 (56.0)	723 (59.6)	739 (60.9)	278 (68)
**Race**	*White*	783 (64.5)	797 (65.7)	819 (67.5)	
	Black	116 (9.6)	120 (9.9)	114 (9.4)	
	Asian	42 (3.5)	34 (2.8)	30 (2.5)	
	Other	162 (13.3)	149 (12.3)	139 (11.4)	
**ECOG, n (%)**	*<2*	588 (48.4)	550 (45.3)	596 (49.1)	399 (98)
	≥2	6 (0.5)	36 (3.0)	131 (10.8)	8 (2)
	Unknown	620 (51.1)	627 (51.7)	487 (40.1)	
**Year of metastatic diagnosis, n (%)**	*2011-2014*	233 (19.2)	227 (18.7)	202 (16.6)	
	2015-2018	565 (46.5)	578 (47.7)	550 (45.3)	0 (0)
	2019-2022	416 (34.3)	408 (33.6)	462 (38.1)	0 (0)
**Site of primary cancer, n (%)**	*Colon, left*	408 (33.6)	286 (23.6)	247 (20.3)	
	Colon, right	198 (16.3)	280 (23.1)	381 (31.4)	241 (59)
	Colon, unknown	314 (25.9)	341 (28.1)	326 (26.9)	
	Rectum	274 (22.6)	273 (22.5)	219 (18.0)	148 (36)
	Unknown	20 (1.6)	33 (2.7)	41 (3.4)	3 (1)
**Labs at start of treatment, n (%)**	*WBC ≥8 x 10^9 per L*	261 (22.8)	403 (36.5)	647 (56.0)	169 (42)
	ALK ≥300 U/L	29 (2.5)	55 (5.0)	236 (20.4)	49 (12)
	Creatinine >2 mg/dl	2 (0.2)	9 (0.8)	26 (2.2)	0 (0)
	Hemoglobin <9 g/dl	26 (2.3)	53 (4.8)	125 (10.8)	0 (0)
	Total bilirubin >3 mg/dl	4 (0.3)	1 (0.1)	39 (3.4)	0 (0)
**Time to death from diagnosis (days), median**		955	710	359	

Supplementary Table 12: Baseline characteristics of the patients in the emulated FIRE-3 trial from the primary analysis, stratified by risk phenotype. The RCT column displays the baseline values of participants reported in Table 1 of the published trial.

Cancer	Trial	Probability of Survival (95% CI)											
		Low-risk			Med-risk			High-risk			RCT		
		Treat. Arm	Cont. Arm	Δ Arms	Treat. Arm	Cont. Arm	Δ Arms	Treat. Arm	Cont. Arm	Δ Arms	Treat. Arm	Cont. Arm	Δ Arms
aNSCLC	KEYNOTE-189	49.1 (44.6-53.8)	43.7 (42.4-45.0)	5.4	27.6 (23.1-31.9)	24.8 (23.8-26.0)	2.7	13.9 (10.8-16.9)	9.0 (8.3-9.9)	4.8	45.7	27.3	18.4
	CHECKMATE-078	52.6 (49.7-55.8)	37.2 (29.0-45.9)	15.4	42.6 (39.6-45.4)	29.6 (17.4-40.8)	13.0	26.9 (24.3-29.5)	10.6 (5.8-18.0)	16.3	50.0	39.0	11.0
	KEYNOTE-024	44.6 (40.3-48.6)	36.9 (27.9-46.5)	7.7	28.0 (23.7-32.2)	27.9 (18.2-39.0)	0.1	15.3 (12.0-18.6)	6.5 (2.2-11.7)	8.7	48.0	15.0	0.33
	KEYNOTE-042	50.1 (45.0-55.6)	52.2 (45.9-57.2)	-2.1	38.4 (33.3-43.4)	34.7 (29.5-41.0)	3.7	19.3 (15.5-23.3)	12.2 (9.1-15.5)	7.1	42.0	28.0	14.0

Supplementary Table 13: Probability of survival for treatment (Treat.) and control (Cont.) arms for a variety of emulated immunotherapy trials from the primary analysis, stratified by prognostic risk phenotypes and involving patients with advanced non-small cell lung cancer (aNSCLC). Probability of survival is calculated at 2 years for KEYNOTE-189 and KEYNOTE-042, and at 1 year for CHECKMATE-078. In KYENOTE-024, probability of progression-free survival is reported at 1 year. The difference in probability of survival between treatment and control arms is labelled Δ Arm.

Cancer	Trial	Patient Population	Treatment Arm	Control Arm	Primary Outcome	Treatment Arm, months (95% CI)	Control Arm, months (95% CI)
aNSCLC	**FLAURA**	Untreated EGFR-mutated advanced disease	Osimertinib	Gefitinib or erlotinib	mPFS	18.9 (15.2-21.4)	10.2 (9.6-11.1)
	**KEYNOTE-189**	Untreated advanced disease without sensitizing EGFR or ALK mutations	Pembrolizumab + Chemotherapy	Placebo + Chemotherapy	mOS	22.0 (19.5-24.5)	10.6 (8.7-13.6)
	**CHECKMATE-078**	Advanced disease previously treated with chemotherapy and without sensitizing EGFR or ALK mutations	Nivolumab	Docetaxel	mOS	12.0 (10.4-14.0)	9.6 (7.6-11.2)
	**KEYNOTE-024**	Untreated advanced disease with PDL1 ≥50% and without sensitizing EGFR or ALK mutations	Pembrolizumab	Chemotherapy	mPFS	10.3 (6.7-n/a)	6.0 (4.2-6.2)
	**KEYNOTE-042**	Untreated advanced disease with PDL1 ≥1% and without sensitizing EGFR or ALK mutations	Pembrolizumab	Chemotherapy	mOS	16.7 (13.9-19.7)	12.1 (11.3-13.3)
mBC	**PALOMA-2**	Untreated advanced disease and estrogen receptor-positive/HER2-negative	Palbociclib + Letrozole	Placebo + Letrozole	mPFS	27.6 (22.4-30.3)	14.5 (12.3-17.1)
	**PALOMA-3**	Advanced disease progressed after previous endocrine therapy and hormone receptor-positive/HER2-negative	Palbociclib + Fulvestrant	Placebo + Fulvestrant	mPFS	9.5 (9.2-11.0)	4.6 (3.5-5.6)
	**CLEOPATRA**	Untreated metastatic disease and HER2-positive	Pertuzumab + Trastuzumab and Docetaxel	Placebo + Trastuzumab and Docetaxel	mOS	57.1 (50.0-72.0)	40.8 (36.0-48.0)
mPC	**CHAARTED**	Untreated metastatic disease and hormone-sensitive	Docetaxel + ADT	Placebo + ADT	mOS	57.6	44.0
	**LATITUDE**	Untreated metastatic disease and hormone-sensitive	Abiraterone + ADT	Placebo + ADT	mOS	53.3 (48.2-n/a)	36.5 (33.5-40.0)
mCRC	**FIRE-3**	Untreated metastatic disease and KRAS wild-type	Cetuximab + FOLFIRI	Bevacizumab + FOLFIRI	mOS	33.1 (24.5-39.4)	25.6 (22.7-28.6)

Supplementary Table 14: Summary of RCT results in patients with advanced non-small cell lung cancer (aNSCLC), metastatic breast cancer (mBC), metastatic prostate cancer (mPC), or metastatic colorectal cancer (mCRC). Key abbreviations: ADT (androgen deprivation therapy), mOS (median overall survival) and mPFS (median progression-free survival).

Feature names for aNSCLC models (total = 133)
**Demographics and cancer characteristics**
'PracticeType'
'gender'
'age'
'Histology,
'SmokingStatus'
'stage'
'adv_year'
'delta_adv_diagnosis'
'cns_met'
'bone_met'
'liver_met'
'resp_met'
'adrenal_met'
'other_met'
**Medications**
'steroid_diag'
'opioid_PO_diag’
'nonopioid_PO_diag'
'pain_IV_diag'
'ac_diag'
'antiinfective_IV_diag'
'antiinfective_diag'
'antihyperglycemic_diag'
'ppi_diag'
'antidepressant_diag'
'bta_diag'
'thyroid_diag'
'is_diag'
**Biomarkers**
'ALK'
'BRAF'
'EGFR'
'KRAS'
'ROS1'
'pdl1'
'pdl1_n'
**Performance status and weight**
'ecog_diagnosis'
'weight_diag'
'bmi_diag'
'bmi_diag_na'
'weight_pct_change'
'weight_pct_na'
'weight_slope'
**Labs**
'albumin_diag'
'alp_diag'
'alt_diag'
'ast_diag'
'bicarb_diag'
'bun_diag'

'calcium_diag'
'chloride_diag'
'creatinine_diag'
'hemoglobin_diag'
'neutrophil_count_diag'
'platelet_diag'
'potassium_diag'
'sodium_diag'
'total_bilirubin_diag'
'wbc_diag'
'albumin_diag_na'
'alp_diag_na'
'alt_diag_na'
'ast_diag_na'
'bicarb_diag_na'
'bun_diag_na'
'calcium_diag_na'
'chloride_diag_na'
'creatinine_diag_na'
'hemoglobin_diag_na'
'neutrophil_count_diag_na'
'platelet_diag_na'
'potassium_diag_na'
'sodium_diag_na'
'total_bilirubin_diag_na'
'wbc_diag_na'
'alp_max'
'alt_max'
'ast_max'
'calcium_max'
'creatinine_max'
'total_bilirubin_max'
'wbc_max'
'albumin_min'
'bicarb_min'
'hemoglobin_min'
'platelet_min'
'sodium_min'
'wbc_min'
'alp_max_na'
'alt_max_na'
'ast_max_na'
'calcium_max_na'
'creatinine_max_na'
'total_bilirubin_max_na'
'wbc_max_na'
'albumin_min_na'
'bicarb_min_na'
'hemoglobin_min_na'
'platelet_min_na'
'sodium_min_na'
'wbc_min_na'
**Past medical history**
'chf'
'cardiac_arrhythmias'

'valvular_disease'
'pulmonary_circulation'
'peripheral_vascular'
'htn_uncomplicated'
'htn_complicated'
'paralysis'
'other_neuro_disorders'
'chronic_pulmonary'
'diabetes_uncomplicated'
'diabetes_complicated'
'hypothyroidism'
'renal_failure'
'liver_disease'
'peptic_ulcer_disease'
'aids_hiv'
'lymphoma'
'metastatic_cancer'
'solid_tumor_wout_mets'
'rheumatoid_arthritis'
'coagulopathy'
'obesity'
'weight_loss'
'fluid_electrolyte'
'blood_loss_anemia'
'deficiency_anemia'
'alcohol_abuse'
'drug_abuse'
'psychoses'
'depression'
'elixhauser_other'
'icd_count'
'other_cancer'

Supplementary Table 15: List of feature names used in the advanced non-small cell lung cancer (aNSCLC) models.

Feature names for mBC models (total = 132)
**Demographics and cancer characteristics**
'gender'
'age'
'practice_type'
'stage'
'met_year'
'delta_met_diagnosis'
'bone_met'
'thorax_met'
'lymph_met'
'liver_met'
'cns_met'
'skin_met'
'peritoneum_met'
'other_met'
**Medications**
'steroid_diag'
'opioid_PO_diag'
'nonopioid_PO_diag'
'pain_IV_diag'
'ac_diag'
'antiinfective_IV_diag'
'antiinfective_diag'
'antihyperglycemic_diag'
'ppi_diag'
'antidepressant_diag'
'bta_diag'
'thyroid_diag'
'is_diag'
**Biomarkers**
'ER'
'HER2'
'PR'
'BRCA'
'PIK3CA'
'pdl1_n'
**Performance status and weight**
'ecog_diagnosis'
'weight_diag'
'bmi_diag'
'bmi_diag_na'
'weight_pct_change'
'weight_pct_na'
'weight_slope'
**Labs**
'albumin_diag'
'alp_diag'
'alt_diag'
'ast_diag'
'bicarb_diag'
'bun_diag'
'calcium_diag'
'chloride_diag'

'creatinine_diag'
'hemoglobin_diag'
'neutrophil_count_diag'
'platelet_diag'
'potassium_diag'
'sodium_diag'
'total_bilirubin_diag'
'wbc_diag'
'albumin_diag_na'
'alp_diag_na'
'alt_diag_na'
'ast_diag_na'
'bicarb_diag_na'
'bun_diag_na'
'calcium_diag_na'
'chloride_diag_na'
'creatinine_diag_na'
'hemoglobin_diag_na'
'neutrophil_count_diag_na'
'platelet_diag_na'
'potassium_diag_na'
'sodium_diag_na'
'total_bilirubin_diag_na'
'wbc_diag_na'
'alp_max'
'alt_max'
'ast_max'
'calcium_max'
'creatinine_max'
'total_bilirubin_max'
'wbc_max'
'albumin_min'
'bicarb_min'
'hemoglobin_min'
'platelet_min'
'sodium_min'
'wbc_min'
'alp_max_na'
'alt_max_na'
'ast_max_na'
'calcium_max_na'
'creatinine_max_na'
'total_bilirubin_max_na'
'wbc_max_na'
'albumin_min_na'
'bicarb_min_na'
'hemoglobin_min_na'
'platelet_min_na'
'sodium_min_na'
'wbc_min_na'
**Past medical history**
'chf'
'cardiac_arrhythmias'
'valvular_disease'
'pulmonary_circulation'

'peripheral_vascular'
'htn_uncomplicated'
'htn_complicated'
'paralysis'
'other_neuro_disorders'
'chronic_pulmonary'
'diabetes_uncomplicated'
'diabetes_complicated'
'hypothyroidism'
'renal_failure'
'liver_disease'
'peptic_ulcer_disease'
'aids_hiv'
'lymphoma'
'metastatic_cancer'
'solid_tumor_wout_mets'
'rheumatoid_arthritis'
'coagulopathy'
'obesity'
'weight_loss'
'fluid_electrolyte'
'blood_loss_anemia'
'deficiency_anemia'
'alcohol_abuse'
'drug_abuse'
'psychoses'
'depression'
'elixhauser_other'
'icd_count'
'other_cancer'

Supplementary Table 16: List of feature names used in the metastatic breast cancer (mBC) models.

Feature names for mPC models (total = 142)
**Demographics and cancer characteristics**
'age'
'p_type'
'NStage'
'MStage'
'Histology'
'GleasonScore'
'stage'
'met_year'
'delta_met_diagnosis'
'crpc'
'crpc_time'
'thorax_met'
'peritoneum_met'
'liver_met'
'other_gi_met'
'cns_met'
'bone_met'
'lymph_met'
'kidney_bladder_met'
'other_met'
'prim_treatment'
'early_adt'
**Medications**
'steroid_diag'
'opioid_PO_diag'
'nonopioid_PO_diag'
'pain_IV_diag'
'ac_diag'
'antiinfective_IV_diag'
'antiinfective_diag'
'antihyperglycemic_diag'
'ppi_diag'
'antidepressant_diag'
'bta_diag'
'thyroid_diag'
**Biomarkers**
'brca_status'
**Performance status and weight**
'ecog_diagnosis'
'weight_diag'
'bmi_diag'
'bmi_diag_na'
'weight_pct_change'
'weight_pct_na'
'weight_slope'
**Labs**
'PSADiagnosis'
'PSAMetDiagnosis'
'albumin_diag'
'alp_diag'
'alt_diag'

'ast_diag'
'bicarb_diag'
'bun_diag'
'calcium_diag'
'chloride_diag'
'creatinine_diag'
'hemoglobin_diag'
'neutrophil_count_diag'
'platelet_diag'
'potassium_diag'
'sodium_diag'
'total_bilirubin_diag'
'wbc_diag'
'albumin_diag_na'
'alp_diag_na'
'alt_diag_na'
'ast_diag_na'
'bicarb_diag_na'
'bun_diag_na'
'calcium_diag_na'
'chloride_diag_na'
'creatinine_diag_na'
'hemoglobin_diag_na'
'neutrophil_count_diag_na'
'platelet_diag_na'
'potassium_diag_na'
'sodium_diag_na'
'total_bilirubin_diag_na'
'wbc_diag_na'
'alp_max'
'alt_max'
'ast_max'
'calcium_max'
'creatinine_max'
'psa_max'
'total_bilirubin_max'
'wbc_max'
'albumin_min'
'bicarb_min'
'hemoglobin_min'
'platelet_min'
'sodium_min'
'wbc_min'
'psa_slope'
'psa_diag_na'
'psa_met_na'
'psa_slope_na'
'alp_max_na'
'alt_max_na'
'ast_max_na'
'calcium_max_na'
'creatinine_max_na'
'psa_max_na'
'total_bilirubin_max_na'
'wbc_max_na'

'albumin_min_na'
'bicarb_min_na'
'hemoglobin_min_na'
'platelet_min_na'
'sodium_min_na'
'wbc_min_na'
**Past medical history**
'chf'
'cardiac_arrhythmias'
'valvular_disease'
'pulmonary_circulation'
'peripheral_vascular'
'htn_uncomplicated'
'htn_complicated'
'paralysis'
'other_neuro_disorders'
'chronic_pulmonary'
'diabetes_uncomplicated'
'diabetes_complicated'
'hypothyroidism'
'renal_failure'
'liver_disease'
'peptic_ulcer_disease'
'aids_hiv'
'lymphoma'
'metastatic_cancer'
'solid_tumor_wout_mets'
'rheumatoid_arthritis'
'coagulopathy'
'obesity'
'weight_loss'
'fluid_electrolyte'
'blood_loss_anemia'
'deficiency_anemia'
'alcohol_abuse'
'drug_abuse'
'psychoses'
'depression'
'elixhauser_other'
'icd_count'
'other_cancer'

Supplementary Table 17: List of feature names used in the metastatic prostate cancer (mPC) models.

Feature names for mCRC models (total = 133)
**Demographics and cancer characteristics**
'gender
'age'
'practice_type'
'stage'
'met_year'
'delta_met_diagnosis'
'crc_site'
'adjuv'
'thorax_met'
'peritoneum_met'
'liver_met'
'other_gi_met'
'cns_met'
'bone_met'
'other_met'
**Medications**
'steroid_diag'
'opioid_PO_diag'
'nonopioid_PO_diag'
'pain_IV_diag'
'ac_diag'
'antiinfective_IV_diag'
'antiinfective_diag'
'antihyperglycemic_diag'
'ppi_diag'
'antidepressant_diag'
'bta_diag'
'thyroid_diag'
'is_diag'
**Biomarkers**
'KRAS'
'dMMR_MSIh'
'NRAS'
'BRAF'
**Performance status and weight**
'ecog_diagnosis'
'weight_diag'
'bmi_diag'
'bmi_diag_na'
'weight_pct_change'
'weight_pct_na'
'weight_slope'
**Labs**
'albumin_diag'
'alp_diag'
'alt_diag'
'ast_diag'
'bicarb_diag'
'bun_diag'
'calcium_diag'
'cea_diag'

'chloride_diag'
'creatinine_diag'
'hemoglobin_diag'
'neutrophil_count_diag'
'platelet_diag'
'potassium_diag'
'sodium_diag'
'total_bilirubin_diag'
'wbc_diag'
'albumin_diag_na'
'alp_diag_na'
'alt_diag_na'
'ast_diag_na'
'bicarb_diag_na'
'bun_diag_na'
'calcium_diag_na'
'cea_diag_na'
'chloride_diag_na'
'creatinine_diag_na'
'hemoglobin_diag_na'
'neutrophil_count_diag_na'
'platelet_diag_na'
'potassium_diag_na'
'sodium_diag_na'
'total_bilirubin_diag_na'
'wbc_diag_na'
'alp_max'
'alt_max'
'ast_max'
'cea_max'
'creatinine_max'
'total_bilirubin_max'
'wbc_max'
'albumin_min'
'bicarb_min'
'hemoglobin_min'
'platelet_min'
'sodium_min'
'wbc_min'
'alp_max_na'
'alt_max_na'
'ast_max_na'
'cea_max_na'
'creatinine_max_na'
'total_bilirubin_max_na'
'wbc_max_na'
'albumin_min_na'
'bicarb_min_na'
'hemoglobin_min_na'
'platelet_min_na'
'sodium_min_na'
'wbc_min_na'
**Past medical history**
'chf'
'cardiac_arrhythmias'

'valvular_disease'
'pulmonary_circulation'
'peripheral_vascular'
'htn_uncomplicated'
'htn_complicated'
'paralysis'
'other_neuro_disorders'
'chronic_pulmonary'
'diabetes_uncomplicated'
'diabetes_complicated'
'hypothyroidism'
'renal_failure'
'liver_disease'
'peptic_ulcer_disease'
'aids_hiv'
'lymphoma'
'metastatic_cancer'
'solid_tumor_wout_mets'
'rheumatoid_arthritis'
'coagulopathy'
'obesity'
'weight_loss'
'fluid_electrolyte'
'blood_loss_anemia'
'deficiency_anemia'
'alcohol_abuse'
'drug_abuse'
'psychoses'
'depression'
'elixhauser_other'
'icd_count'
'other_cancer'

Supplementary Table 18: List of feature names used in the metastatic colorectal cancer (mCRC) models.

Model	Hyperparameters	Description	Search space
GBM	n_estimators	Number of regression tress	Early stopping – training stops once the validation t-AUC reaches a maximum
	learning_rate	Shrinks the contribution of each regression tree	Tuned to achieve at least 500 regression trees with early stopping
	max_depth	Maximum depth of the individual regression trees	[2, 3, 4]
	subsample	The fraction of samples to be used for fitting the regression trees	[0.5, 0.75, 0.9]
RSF	n_estimators	Number of regression tress	Early stopping – training stops once the validation t-AUC reaches a maximum
	max_features	The number of features to consider when looking for the best split	[0.1, 0.333, 0.5, 0.75, 0.9]
	min_samples_leaf	The minimum number of samples required to be a leaf node.	[5, 10, 15]
SVM	alpha	Weight of penalizing square hinge loss in the objective function	10 ** np.linspace(-3, 3, 15)
pCOX, ridge	alpha	Regularization parameter	10 ** np.linspace(-4, 4, 25)
pCOX, lasso	alpha	Regularization parameter	100 alphas along regularization path
pCOX, elastic net	alpha	Regularization parameter	100 alphas along regularization path

Supplementary Table 19: Hyperparameter grid search space. Other hyperparameters were left as default. Key abbreviations are as follows: GBM (Gradient Boosting Model), pCOX (Penalized Cox), RSF (Random Survival Forest), SVM (Support Vector Machines), t-AUC (time-dependent area under the receiver operating characteristic curve).

FLAURA Criteria	Primary	Strict	Not Met
Male or female, aged at least 18 years.	X	X	
Pathologically confirmed adenocarcinoma of the lung.	X	X	
Locally advanced or metastatic NSCLC, not amenable to curative surgery or radiotherapy.	X	X	
The tumour harbours one of the 2 common EGFR mutations known to be associated with EGFR-TKI sensitivity (Ex19del, L858R).	X	X	
Patients must be treatment-naïve for locally advanced or metastatic NSCLC and eligible to receive first-line treatment with gefitinib or erlotinib as selected by the participating centre. Prior adjuvant and neo-adjuvant therapy is permitted(chemotherapy, radiotherapy, investigational agents).	X	X	
Received first line osimertinib or gefitinib or erlotinib	X	X	
No prior treatment with any systemic anti-cancer therapy for locally advanced/metastatic NSCLC.	X	X	
No prior treatment with an EGFR-TKI.	X	X	
World Health Organization Performance Status of 0 to 1 with no clinically significant deterioration over the previous 2 weeks and a minimum life expectancy of 12 weeks		X	
Adequate organ function		X	
No spinal cord compression, symptomatic and unstable brain metastases, except for those patients who have completed definitive therapy, are not on steroids, have a stable neurologic status for at least 2 weeks after completion of the definitive therapy and steroids.		X	
No evidence of severe or uncontrolled systemic diseases, including uncontrolled hypertension and active bleeding diatheses; or active infection including hepatitis B, hepatitis C and human immunodeficiency virus (HIV).		X	
No past medical history of ILD, drug-induced ILD, radiation pneumonitis which required steroid treatment, or any evidence of clinically active ILD.		X	
No major surgery within 4 weeks of the first dose of study drug.			X
No radiotherapy treatment to more than 30% of the bone marrow or with a wide field of radiation within 4 weeks of the first dose of study drug.			X
No patients currently receiving medications or herbal supplements known to be potent inducers of cytochrome P450 (CYP) 3A4.			X
No concurrent and/or other active malignancy that has required treatment within 2 years of first dose of study drug.			X
No refractory nausea and vomiting, chronic gastrointestinal diseases, inability to swallow the formulated product, or previous significant bowel resection that would preclude adequate absorption of AZD9291.			X
No mean resting corrected QT interval (QTc) >470 msec, obtained from 3 ECGs, using the screening clinic ECG machine-derived QTcF value.			X
No clinically important abnormalities in rhythm, conduction, or morphology of resting ECG.			X
No risk factors that increase the risk of QTc prolongation or risk of arrhythmic events or unexplained sudden death under 40 years of age in first-degree relatives or any concomitant medication known to prolong the QT interval.			X

Supplementary Table 20: FLAURA eligibility criteria reproduced for primary and strict emulated trials.

KEYNOTE-189 Criteria	Primary	Strict	Not Met
Has a histologically-confirmed or cytologically confirmed diagnosis of stage IV nonsquamous NSCLC.	X	X	
Has confirmation that epidermal growth factor receptor (EGFR) or anaplastic lymphoma kinase (ALK)-directed therapy is not indicated.	X	X	
Has not received prior systemic treatment for their advanced/metastatic NSCLC.	X	X	
Received first line pemobrolizumab plus platinum-based chemotherapy (carboplatin + pemetrexed or cisplatin + pemetrexed) or platinum-based chemotherapy	X	X	
Before the first dose of study medication: a) Has not received prior systemic cytotoxic chemotherapy for metastatic disease, b) Has not received antineoplastic biological therapy (e.g., erlotinib, crizotinib, cetuximab), c) Has not had major surgery (<3 weeks prior to first dose)	X	X	
Is not currently participating and receiving study therapy or has participated in a study of an investigational agent and received study therapy or used an investigational device within 4 weeks prior to administration of pembrolizumab.	X	X	
ECOG 0 or 1		X	
Adequate organ function		X	
Does not have known active central nervous system (CNS) metastases and/or carcinomatous meningitis.		X	
Does not have active autoimmune disease that has required systemic treatment in past 2 years.		X	
Does not have a known history of Human Immunodeficiency Virus (HIV).		X	
Does not have a known history of active Hepatitis B or C.		X	
Does not have known psychiatric or substance abuse disorder that would interfere with cooperation with the requirements of the trial.		X	
Is not a regular user (including "recreational use") of any illicit drugs or had a recent history (within the last year) of substance abuse (including alcohol).		X	
Does not have interstitial lung disease or a history of pneumonitis that required oral of IV glucocorticoids to assist with management.		X	
Life expectancy of at least 3 months			X
If female of childbearing potential, is willing to use adequate contraception for the course of the study through 120 days after the last dose of study medication or through 180 days after last dose of chemotherapeutic agents.			X
If male with a female partner(s) of child-bearing potential, must agree to use adequate contraception starting with the first dose of study medication through 120 days after the last dose of study medication or through 180 days after last dose of chemotherapeutic agents.			X
Does not have predominantly squamous cell histology NSCLC.			X
Has not received radiation therapy to the lung that is >30 Gray (Gy) within 6 months of the first dose of study medication.			X
Has not completed palliative radiotherapy within 7 days of the first dose of study medication.			X
Has not received a live-virus vaccination within 30 days of planned start of study medication.			X
Does not have a clinically active diverticulitis, intra-abdominal abscess, gastrointestinal obstruction, peritoneal carcinomatosis.			X
Does not have a known history of prior malignancy except if participant has undergone potentially curative therapy with no evidence of that disease recurrence for 5 years since initiation of that therapy, except for successful definitive resection of basal cell carcinoma of the skin, superficial bladder cancer, squamous cell carcinoma of the skin, in situ cervical cancer, or other in situ cancers.			X
Has not previously had a severe hypersensitivity reaction to treatment with another monoclonal antibody (mAb).			X
Does not have a known sensitivity to any component of cisplatin, carboplatin or pemetrexed.			X
Is not on chronic systemic steroids.			X

Is able to interrupt aspirin or other nonsteroidal anti-inflammatory drugs (NSAIDs), other than an aspirin dose ≤1.3 g per day, for a 5-day period (8-day period for long-acting agents, such as piroxicam).			X
Is able or willing to take folic acid or vitamin B12 supplementation.			X
Had not had prior treatment with any other anti-programmed cell death-1 (PD-1), or PD-ligand 1 (PD-L1) or PD-L2 agent or an antibody targeting other immuno-regulatory receptors or mechanisms. Has participated in any other pembrolizumab study and has been treated with pembrolizumab.			X
Does not have an active infection requiring therapy.			X
Does not have symptomatic ascites or pleural effusion.			X
Is not pregnant or breastfeeding, or expecting to conceive or father children prior to 120 days after the last dose of study medication or through 180 days after last dose of chemotherapeutic agents.			X

Supplementary Table 21: KEYNOTE-189 eligibility criteria reproduced for primary and strict emulated trials.

CHECKMATE-078 Criteria	Primary	Strict	Not Met
Disease progression experienced during or after one prior platinum containing doublet chemotherapy	X	X	
Stage IIIb/IV or recurrent disease	X	X	
Male and Female ≥ 18 years of age	X	X	
No prior treatment with Docetaxel	X	X	
No prior treatment with ipilimumab or any drug targeting T-Cell costimulation or checkpoint pathways	X	X	
No EGFR mutation or ALK translocation	X	X	
ECOG ≤ 1		X	
No active central nervous system (CNS) metastases		X	
No history of auto immune diseases		X	
No history of carcinomatous meningitis		X	
No abnormal organ function		X	
Measurable disease per RECIST 1.1			X

Supplementary Table 22: CHECKMATE-078 eligibility criteria reproduced for primary and strict emulated trials. In the RCT, 8% of participants received an EGFR or ALK inhibitor after receiving therapy of interest. However, in the emulated trial, participants were not allowed to receive EGFR or ALK inhibitor therapy after disease progression.

KEYNOTE-024 Criteria	Primary	Strict	Not Met
Stage IV NSCLC lacking epidermal growth factor receptor (EGFR)-sensitizing mutation and/or anaplastic lymphoma kinase (ALK) translocation, and received no prior systemic chemotherapy treatment for their metastatic NSCLC	X	X	
PD-L1 strong expressing tumor as determined by immunohistochemistry (IHC) at a central laboratory	X	X	
Received first line pembrolizumab or platinum-based chemotherapy (carboplatin + paclitaxel, carboplatin + pemetrexed, carboplatin + gemcitabine, cisplatin + pemetrexed, or cisplatin + gemcitabine)	X	X	
Has received systemic therapy for the treatment of their stage IV NSCLC. Completion of treatment with chemotherapy and/or radiation as part of neoadjuvant/adjuvant therapy is allowed as long as therapy was completed at least 6 months prior to the diagnosis of metastatic disease.	X	X	
Currently participating or has participated in a study of an investigational agent or using an investigational device within 4 weeks of first dose of study drug	X	X	
ECOG 0 or 1		X	
Adequate organ function		X	
Has not received Prior therapy with an anti-PD-1, anti-PD-L1, anti-PD-L2, anti-CD137, or anti-cytotoxic T-lymphocyte-associated antigen-4 (CTLA-4) antibody (including ipilimumab or any other antibody or drug specifically targeting T-cell co-stimulation or checkpoint pathways)		X	
Does not have known central nervous system metastases and/or carcinomatous meningitis		X	
Does not have active autoimmune disease that has required systemic treatment in the past 2 years		X	
Does not have interstitial lung disease or history of pneumonitis that has required oral or IV steroids		X	
Does not have known history of human immunodeficiency virus (HIV)		X	
Does not have known active tuberculosis, or hepatitis B or C		X	
Does not have known psychiatric or substance abuse disorders that would interfere with cooperation with the requirements of the study		X	
Life expectancy of at least 3 months			X
At least one radiographically measurable lesion per RECIST 1.1			X
No history of prior malignancy, with the exception of basal cell carcinoma of the skin, superficial bladder cancer, squamous cell carcinoma of the skin, or in situ cervical cancer, or has undergone potentially curative therapy with no evidence of that disease recurrence for 5 years since initiation of that therapy			X
Provided newly obtained formalin fixed tumor tissue from a biopsy of a tumor at the time of or AFTER the diagnosis of metastatic disease has been made AND from a site not previously irradiated			X
Female participants must have a negative pregnancy test at screening if of childbearing potential or be of non-childbearing potential			X
Has not received systemic steroid therapy < 3 days prior to first dose of study drug or receiving any other form of immunosuppressive medication			X
Has not received any prior systemic cytotoxic chemotherapy, biological therapy or major surgery within 3 weeks of the first dose of study therapy; received lung radiation therapy >30 Gy within 6 months of the first dose of study therapy			X
Has not received allogeneic tissue/solid organ transplantation			X
Has not received or will not receive a live vaccine within 30 days prior to the first study therapy (seasonal flu vaccines that do not contain live vaccine are permitted)			X
Does not have active infection requiring intravenous systemic therapy			X

Is not pregnant or breastfeeding, or expecting to conceive or father children during the study and through 120 days after last dose of pembrolizumab or 180 days after last dose of SOC chemotherapy			X

Supplementary Table 23: KEYNOTE-024 eligibility criteria reproduced for primary and strict emulated trials.

**KEYNOTE-042 Criteria**	**Primary**	**Strict**	**Not Met**
Diagnosis of advanced or metastatic NSCLC	X	X	
PD-L1 positive tumor	X	X	
Received first line pembrolizumab or platinum-based chemotherapy (carboplatin + paclitaxel or carboplatin + pemetrexed)	X	X	
No prior systemic chemotherapy for advanced disease	X	X	
EGFR and ALK negative	X	X	
Has not received prior therapy with an anti-PD-1, anti-PD-L1, anti-PD-L2, anti-CD137, or anti-cytotoxic T-lymphocyte-associated antigen-4 (CTLA-4) antibody (including ipilimumab or any other antibody or drug specifically targeting T-cell co-stimulation or checkpoint pathways)	X	X	
ECOG 0 or 1		X	
Adequate organ function		X	
Does not have known central nervous system metastases and/or carcinomatous meningitis		X	
Does not have active autoimmune disease that has required systemic treatment in the past 2 years		X	
Does not have interstitial lung disease or history of pneumonitis that has required oral or IV steroids		X	
Does not have known history of human immunodeficiency virus (HIV)		X	
Does not have known active Hepatitis B or C		X	
Is not a regular user (including "recreational use") of any illicit drugs or had a recent history (within the last year) of substance abuse (including alcohol)		X	
Life expectancy of at least 3 months			X
At least one radiographically measurable lesion per RECIST 1.1			X
Is not receiving steroid therapy <4 days prior to first dose of study therapy			X
Has not received any prior systemic cytotoxic chemotherapy, biological therapy or major surgery within 3 weeks of the first dose of study therapy; received lung radiation therapy >30 Gy within 6 months of the first dose of study therapy			X
Has not received allogeneic tissue/solid organ transplantation			X
Has not received or will not receive a live vaccine within 30 days prior to the first study therapy (seasonal flu vaccines that do not contain live vaccine are permitted)			X
Does not have active infection requiring intravenous systemic therapy			X
Is not pregnant, breastfeeding, or expecting to conceive or father children within the projected duration of the study			X

Supplementary Table 24: KEYNOTE-042 eligibility criteria reproduced for primary and strict emulated trials.

PALOMA-2 Criteria	Primary	Strict	Not Met
Adult women with locoregionally recurrent or metastatic disease not amenable to curative therapy.	X	X	
Confirmed diagnosis of ER positive breast cancer	X	X	
Confirmed diagnosis of HER2 positive disease	X	X	
No prior systemic anti-cancer therapy for advanced ER+ disease.	X	X	
No prior treatment with any CDK 4/6 inhibitor.	X	X	
Received first line palbociclib and letrozole or letrozole alone	X	X	
Postmenopausal women	X	X	
Eastern Cooperative Oncology Group [ECOG] 0-2		X	
Adequate organ and marrow function		X	
No known uncontrolled or symptomatic CNS metastases		X	
Does not have known HIV		X	
Does not have known severe acute or chronic medical or psychiatric condition.		X	
Measurable disease as per Response Evaluation Criterion in Solid Tumors [RECIST] or bone-only disease			X
Patients without advanced, symptomatic, visceral spread that are at risk of life threatening complication in the short term			X
No prior (neo)adjuvant treatment with letrozole or anastrozole with DFI ≤ 12-months from completion of treatment.			X

Supplementary Table 25: PALOMA-2 eligibility criteria reproduced for primary and strict emulated trials.

PALOMA-3 Criteria	Primary	Strict	Not Met
Women 18 years or older with metastatic or locally advanced disease, not amenable to curative therapy	X	X	
Confirmed diagnosis of HR+/HER2- breast cancer	X	X	
Any menopausal status	X	X	
No prior treatment with any CDK inhibitor, fulvestrant, everolimus, or agent that inhibits the PI3K-mTOR pathway	X	X	
Received second (or third) line palbociclib plus fulvestrant or fulvestrant alone	X	X	
Progressed within 12 months from prior adjuvant or progressed within 1 month from prior advanced/metastatic endocrine breast cancer therapy	X	X	
Eastern Cooperative Oncology Group (ECOG) PS 0-1		X	
Adequate organ and marrow function, resolution of all toxic effects of prior therapy or surgical procedures		X	
Patients without extensive advanced/metastatic, symptomatic visceral disease, or known uncontrolled or symptomatic CNS metastases		X	
Does not have known HIV		X	
Does not have known severe acute or chronic medical or psychiatric condition.		X	
On an LHRH agonist for at least 28 days, if pre-/peri-menopausal, and willing to switch to goserelin (Zoladex ®) at time of randomization.			X
Measurable disease defined by RECIST version 1.1, or bone-only disease			X
No major surgery or any anti-cancer therapy within 2 weeks of randomization			X
No prior stem cell or bone marrow transplantation			X
Not using potent CYP3A4 inhibitors or inducers			X

Supplementary Table 26: PALOMA-3 eligibility criteria reproduced for primary and strict emulated trials.

CLEOPATRA Criteria	Primary	Strict	Not Met
Histologically or cytologically confirmed adenocarcinoma of the breast with locally recurrent or metastatic disease, and candidate for chemotherapy. Participants with measurable and non-measurable disease are eligible (locally recurrent disease must not be amenable to resection with curative intent; participants with de novo Stage IV disease are eligible)	X	X	
Human epidermal growth factor receptor 2 (HER2)-positive metastatic breast cancer (MBC)	X	X	
No history of anti-cancer therapy for MBC (with the exception of one prior hormonal regimen for MBC, which must be stopped prior to randomization)	X	X	
Received first line pertuzumab, trastuzumab, and docetaxel/paclitaxel or first line trastuzumab + docetaxel/paclitaxel	X	X	
No history of receiving any investigational treatment within 28 days of randomization	X	X	
No history of approved or investigative tyrosine kinase/HER inhibitors for breast cancer in any treatment setting, except trastuzumab used in the neoadjuvant or adjuvant setting	X	X	
Eastern Cooperative Oncology Group (ECOG) performance status (PS) 0 or 1		X	
No current clinical or radiographic evidence of central nervous system (CNS) metastases		X	
No inadequate organ function, as defined in the protocol, within 28 days prior to randomization		X	
Not known current infection with human immunodeficiency virus (HIV), hepatitis B virus (HBV), or hepatitis C virus (HCV)		X	
No current uncontrolled hypertension or unstable angina		X	
No history of myocardial infarction within 6 months of randomization		X	
No history of congestive heart failure (CHF) of any New York Heart Association (NYHA) criteria, or serious cardiac arrhythmia requiring treatment (exception: atrial fibrillation or paroxysmal supraventricular tachycardia)		X	
Left ventricular ejection fraction (LVEF) ≥50 percent (%) at baseline (within 42 days of randomization)			X
For women of childbearing potential and men with partners of childbearing potential, agreement to use a highly effective form of contraception and to continue its use for the duration of study treatment and for at least 7 months after the last dose of study treatment			X
No history of systemic breast cancer treatment in the neo-adjuvant or adjuvant setting with a disease-free interval from completion of the systemic treatment (excluding hormonal therapy) to metastatic diagnosis of less than (<)12 months			X
No history of persistent Grade ≥2 hematologic toxicity resulting from previous adjuvant therapy			X
No current peripheral neuropathy of National Cancer Institute-Common Terminology Criteria for Adverse Events (NCI-CTCAE) Version 3.0, Grade ≥3 at randomization			X
No history of other malignancy within the last 5 years, except for carcinoma in situ of the cervix, basal cell carcinoma or squamous cell carcinoma of the skin that has been previously treated with curative intent			X
No history of exposure to cumulative doses of anthracyclines			X

No history of LVEF decline to below 50% during or after prior trastuzumab neo-adjuvant or adjuvant therapy			X
No current dyspnea at rest due to complications of advanced malignancy, or other diseases that require continuous oxygen therapy			X
No current severe, uncontrolled systemic disease			X
No major surgical procedure or significant traumatic injury within 28 days prior to study treatment start or anticipation of the need for major surgery during the course of study treatment			X
Not pregnant or lactating women			X
No receipt of IV antibiotics for infection within 14 days of randomization			X
Not currently on chronic daily treatment with corticosteroids (excluding inhaled steroids)			X
No known hypersensitivity to any of the study drugs			X

Supplementary Table 27: CLEOPATRA eligibility criteria reproduced for primary and strict emulated trials.

CHAARTED Criteria	Primary	Strict	Not Met
Histologically or cytologically confirmed prostate cancer	X	X	
Metastatic disease	X	X	
On androgen-deprivation therapy for < 120 days	X	X	
No prior hormone therapy in the metastatic setting	X	X	
Castrate-sensitive metastatic disease	X	X	
Received ADT or docetaxel plus ADT for first line metastatic therapy	X	X	
Concurrent antiandrogen therapy (e.g., bicalutamide or flutamide) allowed, but not as sole hormonal therapy	X	X	
Eastern Cooperative Oncology Group (ECOG) performance status (PS) 0-2		X	
Adequate organ function		X	
No active cardiac disease, including the following: active angina, symptomatic congestive heart failure, or myocardial infarction within the past 6 months		X	
Fertile patients must use effective contraception			X
At least 4 weeks since prior major surgery and recovered from all toxicity prior to randomization			X
Prior adjuvant or neoadjuvant hormonal therapy allowed if therapy was discontinued ≥ 12 months ago AND there is no evidence of disease, as defined by 1 of the following: PSA < 0.1 ng/dL after prostatectomy plus hormonal therapy or PSA < 0.5 ng/dL and has not doubled above nadir after radiotherapy plus hormonal therapy.			X
Prior adjuvant or neoadjuvant hormonal therapy allowed if therapy lasted no more than 24 months and last depot injection must have expired by the 24-month mark			X
Prior palliative radiotherapy allowed if commenced within 30 days before starting androgen deprivation			X
Anti-androgen therapy allowed as single-agent therapy ≤ 7 days before medial castration to prevent flare			X
More than 30 days (or 6 half-lives) (whichever is longer) since prior participation in another clinical trial			X
Concurrent participation in nontherapeutic trials allowed			X
Prostate-specific antigen (PSA) level has risen and met criteria for progression from its lowest point between the start of androgen-deprivation therapy and randomization			X
Prior malignancy in the past 5 years except for basal cell or squamous cell carcinoma of the skin.			X
No peripheral neuropathy > grade 1			X
No history of severe hypersensitivity reaction to docetaxel or other drugs formulated with polysorbate 80			X
No prior chemotherapy in adjuvant or neoadjuvant setting			X
No concurrent 5-alpha reductase inhibitors			X
Not simultaneously enrolled on Cancer and Leukemia Group B (CALGB) 90202			X

Supplementary Table 28: CHAARTED eligibility criteria reproduced for primary and strict emulated trials.

LATITUDE Criteria	Primary	Strict	Not Met
Newly diagnosed metastatic prostate cancer within 3 months prior to randomization with histologically or cytologically confirmed adenocarcinoma of the prostate without neuroendocrine differentiation or small cell histology	X	X	
Distant metastatic disease documented by positive bone scan or metastatic lesions on computed tomography (CT) or magnetic resonance imaging (MRI) scan	X	X	
No prior pharmacotherapy, radiation therapy, or surgery for metastatic prostate cancer (the following exception are permitted): up to 3 months of androgen deprivation therapy (ADT) with lutenizing hormone releasing hormone agonists or antagonists or orchiectomy with or without concurrent anti-androgens prior Cycle 1 Day 1; participants may have one course of palliative radiation or surgical therapy to treat symptoms resulting from metastatic disease if it was administered at least 28 days prior to Cycle 1 Day 1)	X	X	
Castration-sensitive metastatic disease	X	X	
Received ADT or abiraterone plus ADT for first-line metastatic therapy	X	X	
Eastern Cooperative Oncology Group (ECOG) performance status grade of 0, 1 or 2		X	
Adequate hematologic, hepatic, and renal function		X	
No active or symptomatic viral hepatitis or chronic liver disease		X	
No known brain metastasis		X	
At least 2 of the following high-risk prognostic factors: Gleason score of greater than or equal to (>=8); presence of 3 or more lesions on bone scan; presence of measurable visceral (excluding lymph node disease) metastasis on CT or MRI Response Evaluation Criteria in Solid Tumors (RECIST) Version 1.1 scan			X
Agrees to protocol-defined use of effective contraception			X
No active infection or other medical condition that would make prednisone use contraindicated			X
No chronic medical condition requiring a higher systemic dose of corticosteroid than 5 mg prednisone per day			X
No pathological finding consistent with small cell carcinoma of the prostate			X

Supplementary Table 29: LATITUDE eligibility criteria reproduced for primary and strict emulated trials.

FIRE-3 Criteria	Primary	Strict	Not Met
KRAS-Wildtype status.	X	X	
Histologically confirmed adenocarcinoma of the colon or rectum.	X	X	
Stage IV disease.	X	X	
Age 18 - 75 years.	X	X	
In- or outpatient treatment.	X	X	
First-line receipt of FOLFIRI plus cetuximab or FOLFIRI plus bevacizumab.	X	X	
No prior treatment directed against the epidermal growth factor receptor (EGFR).	X	X	
No prior treatment with bevacizumab.	X	X	
No prior chemotherapy for colorectal cancer, except for adjuvant chemotherapy dating back > 6 months prior to study entry.	X	X	
ECOG 0-2.		X	
Adequate organ function.		X	
No known or suspected cerebral metastases.		X	
No clinically significant coronary heart disease, myocardial infarction within the last 12 months or high risk of uncontrolled arrhythmia.		X	
No acute or subacute ileus, chronic inflammatory bowel disease or chronic diarrhea.		X	
No uncontrolled hypertension.		X	
No severe proteinuria (nephrotic syndrome).		X	
No known alcohol or drug abuse.		X	
No medical or psychiatric condition which contradicts participation of study.		X	
No bleeding diatheses or coagulopathy.		X	
No arterial thromboembolic events or hemorrhage within 6 months prior to study entry (except tumor bleeding surgically treated by tumor resection).		X	
Patients considered suitable for application of chemotherapy.			X
Estimated life expectancy > 3 months.			X
Measurable index lesion according to RECIST criteria. Evaluation of tumor manifestations ≤ 2 weeks prior to treatment start.			X
Effective contraception.			X
No operations within 4 weeks prior to treatment start. No cytologic biopsies within 1 week prior to treatment start. Operation sequels need to be completely healed. Major operations must not be expected at time of study begin, except for potential secondary resection of liver metastases. In case of secondary resection of liver metastases, bevacizumab must be discontinued 6-8 weeks prior to surgery.			X
No relevant toxicities due to prior medical treatment at time of study entry.			X
No experimental medical treatment within 30 days prior to study entry.			X
No known hypersensitivity reaction to any study medication.			X
No pregnant or breast feeding women (pregnancy needs to be excluded by testing of beta-HCG).			X
No symptomatic peritoneal carcinosis.			X
No severe chronic wounds, ulcers or bone fracture.			X
No full dose anticoagulation.			X
No known DPD-deficiency (special screening not required).			X
No known glucuronidation-deficiency (special screening not required).			X
No medical history of other malignant disease within 5 years prior to study entry, except for basalioma, and in-situ cervical carcinoma if treated with curative intent.			X

Supplementary Table 30: FIRE-3 eligibility criteria reproduced for primary and strict emulated trials.

Trial	Chemotherapy	Dose
KEYNOTE-189	Carboplatin	≥5 AUC
	Cisplatin	≥75 mg/m^2
CHECKMATE-078	Docetaxel	≥75 mg/m^2
KEYNOTE-024	Carboplatin	≥5 AUC
	Cisplatin	≥75 mg/m^2
KEYNOTE-042	Carboplatin	≥5 AUC
	Cisplatin	≥75 mg/m^2
CHAARTED	Docetaxel	≥75 mg/m^2
FIRE-3	Oxaliplatin	≥85 mg/m^2
	Irinotecan	≥125 mg/m^2

Supplementary Table 31: Cutoffs used for defining standard dose of chemotherapeutic agents. Cutoffs were determined based on National Comprehensive Cancer Network guidelines.

a

	1-year AUC
**GBM, original**	0.783
**GBM, excluding holdout**	0.791

b

	Hazard ratio (95% CI) for the entire population
**Training**	0.91 (0.85-0.99)
**Holdout**	0.86 (0.77-0.96)

c

	Restricted Mean Survival Time in Months (95% CI)								
	Low-risk			Med-risk			High-risk		
	Treat. Arm	Cont. Arm	Δ Arms	Treat. Arm	Cont. Arm	Δ Arms	Treat. Arm	Cont. Arm	Δ Arms
**Training**	22.43 (21.54-23.32)	21.45 (21.08-21.81)	0.98 (0.02-1.94)	16.08 (15.30-16.87)	15.45 (15.10-15.80)	0.63 (-0.23-1.49)	9.24 (8.52-9.95)	7.52 (7.27-7.77)	1.72 (0.96-2.47)
**Holdout**	23.52 (22.38-24.65)	21.15 (20.63-21.66)	2.37 (1.12-3.62)	16.81 (15.63-17.98)	15.63 (15.14-16.12)	1.18 (-0.10-2.45)	9.42 (8.49-10.35)	8.14 (7.76-8.53)	1.28 (0.27-2.28)

d

	Median Overall Survival in Months (95% CI)								
	Low-risk			Med-risk			High-risk		
	Treat. Arm	Cont. Arm	Δ Arms	Treat. Arm	Cont. Arm	Δ Arms	Treat. Arm	Cont. Arm	Δ Arms
**Training**	23.9 (22.9-25.0)	20.7 (19.8-21.6)	3.2	12.3 (11.8-12.9)	11.4 (11.0-11.7)	0.9	4.9 (4.7-5.0)	4.6 (4.4-4.8)	0.3
**Holdout**	23.9 (23.3-25.8)	19.7 (18.9-20.8)	4.2	12.9 (12.1-13.4)	11.1 (10.6-11.6)	1.8	4.9 (4.6-5.5)	4.6 (4.4-4.8)	0.3
Difference between training and holdout	0.0	1.0	-1.0	-0.6	0.3	-0.9	0.0	0.0	0.0

Supplementary Table 32: Four tables are presented related to the validation of KEYNOTE-189 results. **Table a** compares the discriminatory performance of gradient boosting models (GBM) on a test set: GBM original (trained on the full cancer cohort, Fig. 2) and GBM excluding the holdout dataset. **Table b** displays the hazard ratio for overall survival with treatment for the entire cohort in both the training and holdout datasets. **Table c** shows the restricted mean survival time at 3 years for both the training and holdout datasets across risk phenotypes. **Table d** presents the median overall survival in months for both the training and holdout datasets across risk phenotypes. Abbreviations: treatment (Treat.), control (Cont.), difference between treatment and control arms (Δ Arms).

a

	2-year AUC
**GBM, original**	0.814
**GBM, excluding holdout**	0.805

b

	Hazard ratio (95% CI) for the entire population
**Training**	0.74 (0.65-0.83)
**Holdout**	0.78 (0.70-0.89)

c

	Restricted Mean Progression-Free Survival Time in Months (95% CI)								
	**Low-risk**			**Med-risk**			**High-risk**		
	**Treat. Arm**	**Cont. Arm**	**Δ Arms**	**Treat. Arm**	**Cont. Arm**	**Δ Arms**	**Treat. Arm**	**Cont. Arm**	**Δ Arms**
**Training**	30.31 (28.59-30.04)	25.13 (23.35-26.91)	5.18 (2.70-7.66)	26.50 (24.34-28.66)	20.49 (19.09-21.89)	6.01 (3.43-8.58)	15.91 (14.22-17.60)	13.87 (12.69-15.05)	2.04 (-0.02-4.10)
**Holdout**	30.56 (28.68-32.44)	24.94 (23.19-26.69)	5.62 (3.05-8.19)	24.47 (22.55-26.39)	21.49 (19.98-23.00)	2.97 (0.55-5.41)	18.40 (16.23-20.56)	14.69 (13.29-16.09)	3.70 (1.12-6.28)

d

	Median Progression-Free Survival in Months (95% CI)								
	**Low-risk**			**Med-risk**			**High-risk**		
	**Treat. Arm**	**Cont. Arm**	**Δ Arms**	**Treat. Arm**	**Cont. Arm**	**Δ Arms**	**Treat. Arm**	**Cont. Arm**	**Δ Arms**
**Training**	31.2 (28.5-35.5)	22.1 (19.6-23.1)	9.0	23.9 (21.0-27.1)	16.6 (14.2-18.1)	7.3	11.0 (9.8-12.3)	9.9 (8.8-11.2)	1.1
**Holdout**	34.4 (31.7-37.7)	21.3 (20.1-22.6)	13.1	20.9 (18.4-22.4)	17.3 (16.0-18.7)	3.6	12.6 (10.8-13.5)	10.0 (8.0-10.8)	2.6
Difference between training and holdout	-3.2	0.8	-4.1	3.0	-0.6	3.4	-1.0	-0.1	-1.5

Supplementary Table 33: Four tables are presented related to the validation of PALOMA-2 results. **Table a** compares the discriminatory performance of gradient boosting models (GBM) on a test set: GBM original (trained on the full cancer cohort, Fig. 2) and GBM excluding the holdout dataset. **Table b** displays the hazard ratio for progression-free survival with treatment for the entire cohort in both the training and holdout datasets. **Table c** shows the restricted mean progression-free survival time at 4 years for both the training and holdout datasets across risk phenotypes. **Table d** table presents the median progression-free survival in months for both the training and holdout datasets across risk phenotypes. Abbreviations: treatment (Treat.), control (Cont.), difference between treatment and control arms (Δ Arms).

Sample size	Phenotype	Case 1		Case 2		Case 3		Case 4	
		Coverage	Relative bias	Coverage	Relative bias	Coverage	Relative bias	Coverage	Relative bias
10,000	All	95.10%	2.23% ± 0.04%	34.55%	6.69% ± 0.06%	12.65%	9.00% ± 0.06%	27.60%	6.99% ± 0.06%
	Low	94.75%	4.18% ± 0.07%	76.90%	6.69% ± 0.09%	61.05%	8.82% ± 0.11%	82.70%	5.83% ± 0.08%
	Medium	94.35%	4.15% ± 0.07%	77.40%	6.59% ± 0.09%	64.80%	8.52% ± 0.11%	67.90%	7.53% ± 0.10%
	High	95.25%	3.54% ± 0.06%	64.85%	7.14% ± 0.09%	48.80%	9.20% ± 0.10%	54.80%	7.86% ± 0.09%
15,000	All	94.55%	1.82% ± 0.03%	15.10%	6.89% ± 0.05%	3.10%	9.10% ± 0.05%	10.35%	7.00% ± 0.05%
	Low	94.00%	3.53% ± 0.06%	64.15%	6.74% ± 0.08%	42.80%	9.04% ± 0.09%	73.35%	5.59% ± 0.08%
	Medium	94.65%	3.38% ± 0.06%	66.95%	6.48% ± 0.08%	49.60%	8.44% ± 0.09%	55.15%	7.38% ± 0.08%
	High	95.10%	2.93% ± 0.05%	48.90%	7.32% ± 0.08%	30.10%	9.26% ± 0.08%	40.70%	7.72% ± 0.07%
20,000	All	95.65%	1.57% ± 0.03%	6.20%	6.92% ± 0.04%	0.30%	9.16% ± 0.04%	4.35%	7.05% ± 0.04%
	Low	94.60%	2.92% ± 0.05%	53.45%	6.68% ± 0.07%	30.85%	8.96% ± 0.08%	65.80%	5.46% ± 0.07%
	Medium	94.65%	2.96% ± 0.05%	57.90%	6.48% ± 0.07%	35.25%	8.55% ± 0.08%	42.85%	7.42% ± 0.08%
	High	94.80%	2.61% ± 0.04%	37.75%	7.26% ± 0.07%	17.75%	9.43% ± 0.07%	25.90%	7.84% ± 0.06%

Supplementary Table 34: Four cases were considered in the semi-synthetic data simulation, with 2,000 simulations performed for each case and sample size to calculate the coverage of the 95% confidence interval and the mean relative bias of the hazard ratios. In Case 1, both the positivity assumption and the no unmeasured confounder assumption hold. In Case 2, the positivity assumption is violated (30% never receive treatment) while no unmeasured confounder assumption holds. In Case 3, positivity assumption is violated more severely (40% never receive treatment) while no unmeasured confounder assumption holds. In Case 4, positivity assumption holds while no unmeasured confounder assumption is violated.
