# Supplementary material for: Evaluating generalizability of oncology trial results to real-world patients using machine learning-based trial emulations
Source: Nat Med. 2025 Jan 3;31(2):457–65. doi: 10.1038/s41591-024-03352-5 (PMC11835724; doi:10.1038/s41591-024-03352-5)
Supplement: Supplementary file 2 — Reporting Summary [file 41591_2024_3352_MOESM2_ESM.pdf]

nature portfolio

Corresponding author(s): Ravi Parikh

Last updated by author(s): Sep 27, 2024

Reporting Summary

Nature Portfolio wishes to improve the reproducibility of the work that we publish. This form provides structure for consistency and transparency in reporting. For further information on Nature Portfolio policies, see our Editorial Policies and the Editorial Policy Checklist.

Statistics

For all statistical analyses, confirm that the following items are present in the figure legend, table legend, main text, or Methods section.

n/a	Confirmed	
☐	☒	The exact sample size (*n*) for each experimental group/condition, given as a discrete number and unit of measurement
☐	☒	A statement on whether measurements were taken from distinct samples or whether the same sample was measured repeatedly
☐	☒	The statistical test(s) used AND whether they are one- or two-sided<br>*Only common tests should be described solely by name; describe more complex techniques in the Methods section.*
☐	☒	A description of all covariates tested
☐	☒	A description of any assumptions or corrections, such as tests of normality and adjustment for multiple comparisons
☐	☒	A full description of the statistical parameters including central tendency (e.g. means) or other basic estimates (e.g. regression coefficient) AND variation (e.g. standard deviation) or associated estimates of uncertainty (e.g. confidence intervals)
☐	☒	For null hypothesis testing, the test statistic (e.g. *F*, *t*, *r*) with confidence intervals, effect sizes, degrees of freedom and *P* value noted<br>*Give P values as exact values whenever suitable.*
☒	☐	For Bayesian analysis, information on the choice of priors and Markov chain Monte Carlo settings
☒	☐	For hierarchical and complex designs, identification of the appropriate level for tests and full reporting of outcomes
☐	☒	Estimates of effect sizes (e.g. Cohen's *d*, Pearson's *r*), indicating how they were calculated

Our web collection on statistics for biologists contains articles on many of the points above.

Software and code

Policy information about availability of computer code

Data collection	This study used the nationwide Flatiron Health electronic health record (EHR)-derived database. No software was used for data collection.
Data analysis	Data analysis was conducted using custom Python code (version 3.7) developed by the study authors. The code is open-source and accessible at https://github.com/xavier-orcutt/TrialTranslator-notebooks. Notable Python packages used include scikit-survival (version 0.17) for building the survival machine learning models and miceforest (version 5.6.3) for multiple imputation chained equations. For a complete list of Python packages and their versions, refer to the requirements.txt file in the GitHub repository.

For manuscripts utilizing custom algorithms or software that are central to the research but not yet described in published literature, software must be made available to editors and reviewers. We strongly encourage code deposition in a community repository (e.g. GitHub). See the Nature Portfolio guidelines for submitting code & software for further information.

Data

Policy information about availability of data

All manuscripts must include a data availability statement. This statement should provide the following information, where applicable:

- Accession codes, unique identifiers, or web links for publicly available datasets
- A description of any restrictions on data availability
- For clinical datasets or third party data, please ensure that the statement adheres to our policy

The Flatiron Health data used in this study were made available as part of an academic collaboration with the University of Pennsylvania. The authors had full access

to the data and were responsible for conducting the analyses. This research was conducted in compliance with all relevant ethical regulations. Institutional Review Board approval from the University of Pennsylvania, including a waiver of informed consent, was obtained before the study was conducted. These de-identified data may be made available upon request via a proposal-based process. Interested researchers can contact DataAccess@flatiron.com.

Research involving human participants, their data, or biological material

Policy information about studies with human participants or human data. See also policy information about sex, gender (identity/presentation), and sexual orientation and race, ethnicity and racism.

Reporting on sex and gender	In our manuscript and the webtool, we exclusively used the term "sex" referring to the biological attribute.<br><br>The Flatiron Health data is gathered from EHR-derived patient-level information, likely using sex assigned at birth. However, due to variations in reporting practices across clinical sites, it is unclear whether the sex data is self-reported by patients or recorded by clinicians. Despite this uncertainty, sex was included as a variable in our analysis, specifically in the propensity score model for inverse probability of treatment weighting.<br><br>We suspect our findings apply to both male and female sexes. However, a comprehensive sex-based analysis was not performed since we have no reason to believe that the anticancer agents studied would be significantly influenced by sex.
Reporting on race, ethnicity, or other socially relevant groupings	As previously mentioned, due to variations in reporting practices across clinical sites, it remains unclear whether race and ethnicity data are self-reported by patients or recorded by clinicians. Despite this uncertainty, they were included in the propensity score model for inverse probability of treatment weighting. Additionally, we incorporated a patient's area-level socioeconomic status into the propensity score model. This measure was determined by neighborhood factors including income, home values, rental costs, poverty, unemployment, and education level.<br><br>While these demographic and socioeconomic variables were used for balancing treatment groups, they were deliberately excluded from the machine learning model. This methodological choice aims to balance potential confounding factors across treatment and control arms while mitigating the risk of perpetuating historical or societal biases in the predictive model.
Population characteristics	The Flatiron Health dataset encompassed patients with four types of advanced cancers: non-small cell lung cancer, metastatic breast cancer, metastatic prostate cancer, and metastatic colorectal cancer. Across these cancer types, the median age was approximately 65 years, and the majority of patients were white. Patients were primarily from community cancer centers, with the South being the most represented region. Most patients had private insurance or Medicare, and the majority were diagnosed with advanced disease before 2019. For a more detailed breakdown of patient characteristics specific to each cancer type, please refer to Table 1.
Recruitment	Our study utilized data from the Flatiron Health database, which leverages real-world data collected from EHRs of cancer patients across a network of oncology clinics and academic medical centers in the United States. Patients are not directly recruited; rather, their de-identified data is included based on their cancer diagnosis and treatment at participating healthcare facilities.<br><br>Several potential biases should be acknowledged with the Flatiron Health data. The distribution of participating clinics may not be uniform across the US, potentially over-representing certain regions. Additionally, patients with access to these healthcare facilities may not represent the full spectrum of socioeconomic backgrounds. The data may also be subject to variations in EHR completeness and quality across different facilities.
Ethics oversight	The Institutional Review Board at the University of Pennsylvania granted approval for this study with a waiver of informed consent prior to its commencement.

Note that full information on the approval of the study protocol must also be provided in the manuscript.

Field-specific reporting

Please select the one below that is the best fit for your research. If you are not sure, read the appropriate sections before making your selection.

☒ Life sciences ☐ Behavioural & social sciences ☐ Ecological, evolutionary & environmental sciences

For a reference copy of the document with all sections, see nature.com/documents/nr-reporting-summary-flat.pdf

Life sciences study design

All studies must disclose on these points even when the disclosure is negative.

Sample size	Our study focused specifically on patients with advanced non-small cell lung cancer (aNSCLC), metastatic breast cancer (mBC), metastatic prostate cancer (mPC), and metastatic colorectal cancer (mCRC) from the Flatiron Health Database. The cohort sizes in the Flatiron Health Database were as follows: 68,483 patients with aNSCLC, 31,677 patients with mBC, 18,927 patients with mPC, and 34,315 patients with mCRC. These patients were diagnosed with advanced or metastatic disease between 2011-2022, with about 70% diagnosed before 2019.<br><br>All patients were included in the prognostic model development. For the emulated trials, cohort sizes were determined by patients meeting specific eligibility criteria and varied widely, ranging from 1,200 to 30,000 patients, with an average cohort size of 4,000 patients across trials. Sample sizes for these emulated trials needed to exceed 600 patients to ensure sufficient statistical power. Notably, in all but two of the emulated trials, the sample size within prognostic phenotypes was larger than that of the corresponding RCT.

Data exclusions

All patients in the Flatiron Health Database with aNSCLC, mBC, mPC, and mCRC were included in the prognostic model development. Eligibility for emulated trials in the primary analysis was contingent upon patients meeting 3 criteria: (1) having the correct cancer type, (2) receiving the treatment of interest at the appropriate line of therapy, and (3) possessing relevant biomarker status at the time of treatment.

Replication

The TrialTranslator framework was applied to 4 different cancer types (aNSCLC, mBC, mPC, and mCRC) and across a variety of novel anticancer agents. The robustness of the results was assessed through three types of analyses: sensitivity, validation, and a semi-synthetic data simulation.

In the first sensitivity analysis, the impact of different imputation strategies on the discriminatory performance of the machine learning models was assessed. In the second sensitivity analysis, we investigated whether the treatment effect differed when emulated trials were conducted using strict eligibility criteria instead of the key eligibility criteria from the primary analysis. In the third sensitivity analysis, we investigated whether the treatment effect differed when patient inclusion in the emulated trials was dependent on standard upfront dosing of chemotherapeutic agents, as defined in the National Comprehensive Cancer Network. Each of these analyses was performed independently for each cancer type and anticancer agent combination. All attempts at replication were successful, with consistent patterns observed across cancer types and anticancer agents, although the magnitude of effects varied as expected due to the inherent differences in cancer biology and treatment efficacy.

For the validation assessment, we examined the agreement in treatment effect across risk phenotypes on a holdout set, specifically for KEYNOTE-189 and PALOMA-2.

Lastly, a semi-synthetic data simulation was conducted on KEYNOTE-189 to assess the bias of the trial emulation HRs and the validity of their confidence intervals under various conditions.

Randomization

This study is a retrospective analysis of real-world data, precluding traditional randomization of interventions. Patient allocation to prognostic phenotypes was based on risk scores derived from a machine learning model. Within prognostic phenotypes, inverse propensity treatment weighing was applied to balance measured features between treatment and control arms. This technique aims to mimic the balance of characteristics that would naturally occur in a RCT. These method attempts to address potential confounding and selection bias inherent in observational data, though they cannot fully replicate the benefits of prospective randomization.

Blinding

Patients in this dataset were not research participants in the traditional sense, but rather their existing clinical data was analyzed post-hoc. Therefore, patient blinding to treatment allocation was neither possible nor relevant. Similarly, investigator blinding was not feasible as the data analysis inherently required knowledge of treatment assignments. However, to mitigate potential bias, we employed objective, pre-specified analytical methods and endpoints.

Reporting for specific materials, systems and methods

We require information from authors about some types of materials, experimental systems and methods used in many studies. Here, indicate whether each material, system or method listed is relevant to your study. If you are not sure if a list item applies to your research, read the appropriate section before selecting a response.

Materials & experimental systems

n/a	Involved in the study
☒	☐ Antibodies
☒	☐ Eukaryotic cell lines
☒	☐ Palaeontology and archaeology
☒	☐ Animals and other organisms
☐	☒ Clinical data
☒	☐ Dual use research of concern
☒	☐ Plants

Methods

n/a	Involved in the study
☒	☐ ChIP-seq
☒	☐ Flow cytometry
☒	☐ MRI-based neuroimaging

Clinical data

Policy information about clinical studies
All manuscripts should comply with the ICMJE guidelines for publication of clinical research and a completed CONSORT checklist must be included with all submissions.

Clinical trial registration

This study is a retrospective analysis of real-world data and therefore was not registered as a clinical trial.

Study protocol

Phase III RCTs pertaining to the four cancers were considered for emulation if RCTs demonstrated an overall or progression-free survival benefit for the treatment arm, involved treatment regimens that were standard of care as of January 2023, and the Flatiron Health Database contained at least 600 patients meeting key eligibility criteria for the respective RCT. This selection process resulted in the inclusion of 11 RCTs: 5 for aNSCLC, 3 for mBC, 2 for mPC, and 1 for mCRC

Data collection

This study used the nationwide Flatiron Health EHR-derived Database (https://flatiron.com/real-world-evidence), sourced from approximately 280 cancer clinics across the United States, for both prognostic model development and trial emulation. This database is a longitudinal repository containing de-identified patient-level structured and unstructured data. Features available in this database include baseline demographics, cancer characteristics, biomarkers, past medical history, medications, vital signs, and laboratory values.

Outcomes

For prognostic model development, the primary outcome used to determine the top-perfoming ML model was a time-dependent AUC at 1 year for aNSCLC and 2 years for mBC, mPC, and mCRC. Particular attention was given to these time points since they align with the cancer's median overall survival in the Flatiron Health Dataset.

For the prognostic phenotypes within the emulated trials, the primary outcome was restricted mean survival time and median overall survival. In RCTs where the primary outcome was progression-free survival, restricted mean progression-free survival time and median progression-free survival were calculated instead. In addition, probability of survival at 1 or 2-years from treatment initiation was calculated for trials investigating checkpoint inhibitors (e.g., KEYNOTE-189, CHECKMATE-078, KEYNOTE-024, and KEYNOTE-042), due to their tendency for delayed treatment effects.

Plants

Seed stocks

Report on the source of all seed stocks or other plant material used. If applicable, state the seed stock centre and catalogue number. If plant specimens were collected from the field, describe the collection location, date and sampling procedures.

Novel plant genotypes

Describe the methods by which all novel plant genotypes were produced. This includes those generated by transgenic approaches, gene editing, chemical/radiation-based mutagenesis and hybridization. For transgenic lines, describe the transformation method, the number of independent lines analyzed and the generation upon which experiments were performed. For gene-edited lines, describe the editor used, the endogenous sequence targeted for editing, the targeting guide RNA sequence (if applicable) and how the editor was applied.

Authentication

Describe any authentication procedures for each seed stock used or novel genotype generated. Describe any experiments used to assess the effect of a mutation and, where applicable, how potential secondary effects (e.g. second site T-DNA insertions, mosiacism, off-target gene editing) were examined.
